# Simulating the Energy Capture Process in Push–Pull Norbornadiene-Quadricyclane Photoswitches

**DOI:** 10.1021/acs.jpclett.5c00634

**Published:** 2025-04-23

**Authors:** Michał Andrzej Kochman, Bo Durbeej

**Affiliations:** † Institute of Physical Chemistry of the Polish Academy of Sciences, Ul. Marcina Kasprzaka 44/52, 01-224 Warsaw, Poland; ‡ Theoretical Chemistry, Ruhr University Bochum, Universitätsstraße 150, 44801 Bochum, Germany; ¶ Division of Theoretical Chemistry, Department of Physics, Chemistry and Biology (IFM), Linköping University, 58183 Linköping, Sweden

## Abstract

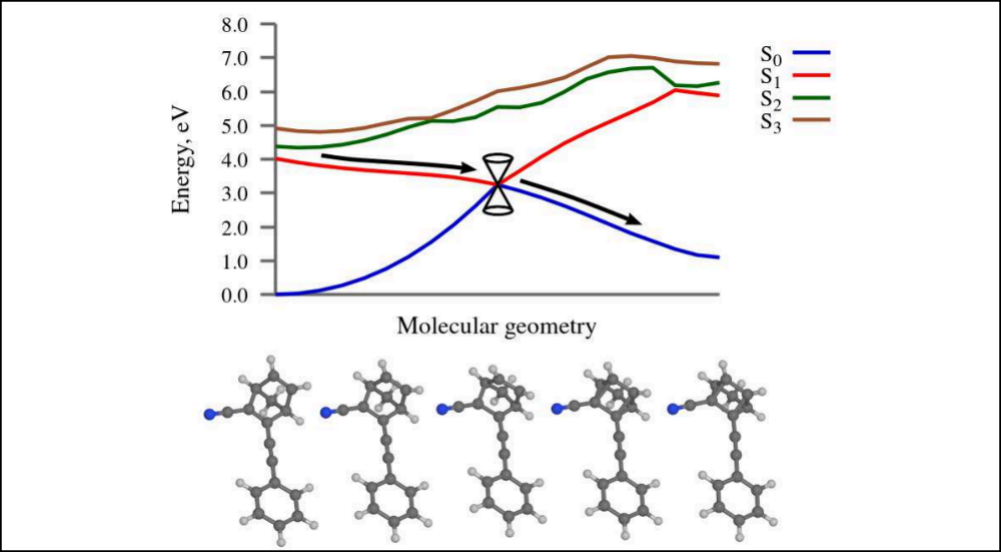

Molecular switches based on the norbornadiene-quadricyclane (NBD-QC) isomer pair are among the most promising candidates for applications in molecular solar thermal energy storage (MOST). In these compounds, solar energy is captured through a photoinduced [2 + 2] cycloaddition reaction whose mechanism is only partially understood. This holds true especially for NBD derivatives containing the type of push–pull substitution pattern that was previously proven necessary to attain reasonable photoisomerization quantum yields. In the present contribution, we report a computational investigation of the photochemistry of NBD-QC switches with precisely such a substitution pattern. Static calculations provide information on the structures of the excited electronic states involved in the photoinduced cycloaddition reaction, and the topographies of the relevant ground- and excited-state potential energy surfaces. Furthermore, nonadiabatic molecular dynamics (NAMD) simulations allow an estimation of the reaction time scale and quantum yield. The simulation results paint a detailed picture of the energy capture process: the photoinduced cycloaddition reaction begins in the spectroscopically bright excited state of the molecular switch. In the model compound for which we performed NAMD simulations, ring closing takes place on a time scale of roughly 150 fs, which makes it one of the fastest known photoisomerization reactions.

Molecular solar thermal energy storage^1−8^ (MOST) is an emerging technology for the collection, storage, and controlled release of energy from solar radiation with the use of molecular switches – compounds which can be reversibly converted between two or more states. In the present study, our focus will be on the norbornadiene-quadricyclane (NBD-QC) system, which is one of the molecular switches which has attracted the most attention so far.^3,9−11^ Its operating cycle is illustrated in Figure 1.

**Figure 1:**
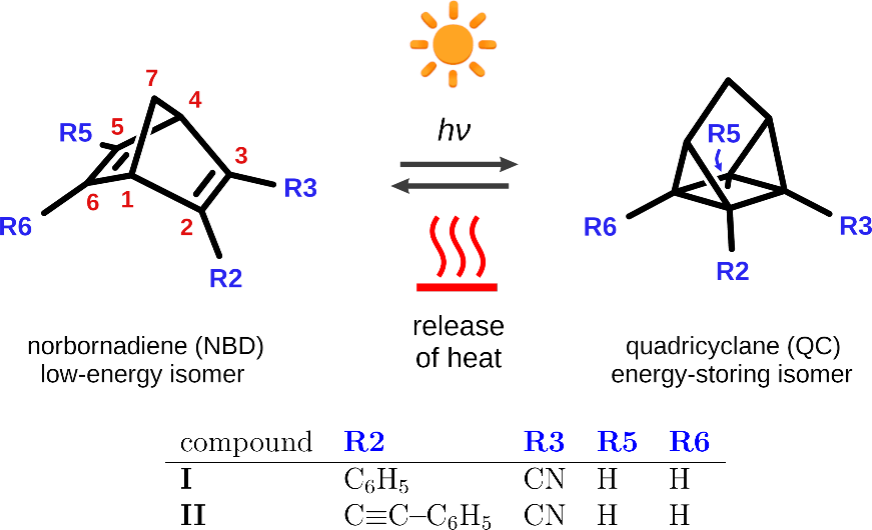
General structure and operating cycle of molecular switches of the NBD-QC class. Atom numbering is shown in red. **R2**, **R3**, **R5** and **R6** are substituents or hydrogen atoms. In the present study, we investigated the photochemistry of compounds **I** and **II**.

The first half of the cycle consists of a photoinduced [2 + 2] cycloaddition reaction, which converts the NBD isomeric form of the switch into the QC form. The QC isomer acts as the energy-storing isomer – due to the presence of two strained three-membered rings, it is significantly higher in energy than the NBD isomer. Crucially, the QC isomer can be stored for an extended period of time. Its long lifetime is a consequence of the Woodward–Hoffmann rules, which predict that thermal [2 + 2] cycloaddition and cycloreversion reactions are impeded by large energy barriers.^12^ (The exact energy difference between the two isomers, and the lifetime of the QC isomer, depend on the choice of substituents.)

In the second half of the operating cycle, the QC isomer undergoes cycloreversion into the NBD form, releasing the stored energy in the form of heat. This reaction is usually triggered with an external stimulus, which can be chemical or electrochemical in nature. For instance, the electrochemical oxidation of a small fraction of the QC isomer to the QC^•+^ radical cation sets off cycloreversion through a radical chain reaction.^13−15^

Aside from the NBD-QC system, other examples of switches with potential applications in MOST include the dihydroazulene-vinylheptafulvene system,^16,17^ derivatives of azobenzene,^18,19^ and switches based on the photoinduced dimerization of anthracene.^20,21^

What sets MOST apart from other technologies for harvesting solar energy is that the capture, storage, and release of energy are all performed by the same material. This feature makes it well suited to applications where energy is to be consumed close to where it is collected, but at a later point in time. Examples include space heating, hot water supply to households, and deicing systems for windows.^22^

However, the fact that the molecular switch plays multiple roles places a number of demands on its physicochemical properties. All potential applications require the maximization of the energy storage density (ESD) per unit weight; an oft-quoted value for the minimum acceptable ESD is 0.3 MJ/kg.^1^ Encouragingly, some existing switches in the NBD-QC series exceed that value, with ESDs of up to around 0.5 MJ/kg.^23,24^ Furthermore, in most cases it is desirable to suppress the spontaneous back-isomerization of the high-energy form of the molecular switch into the low-energy form. This is in order to extend the energy storage time.

Another important requirement is to maximize the rate of energy capture. Here, there are two main factors at play. One is that the low-energy isomer of the switch must strongly absorb solar radiation. (On the other hand, light absorption by the energy-storing isomer is undesirable.) The other is that the efficiency of the photoisomerization reaction through which energy is captured must be as high as possible. Conventionally, the efficiency of photoisomerization is expressed in terms of the quantum yield at a given irradiation wavelength, which is defined as the ratio of photoisomerization events to the number of photons absorbed.

The importance of maximizing photoisomerization quantum yields (and, thereby, the rate of energy capture) has been recognized from the beginnings of research into MOST systems.^25−27^ Yet, with few exceptions,^28−30^ there has been limited progress on understanding the factors which control the quantum yields of the major classes of molecular switches involved.^4^ This point applies in particular to the NBD-QC series. The theoretical studies to date have mainly dealt with the mechanism of photoisomerization in unsubstituted norbornadiene^31−34^ (the prototypical compound in which **R2**, **R3**, **R5** and **R6** are all hydrogen atoms). While interesting for its own sake, unsubstituted norbornadiene is not necessarily a realistic model of practically relevant molecular switches – for that compound, the quantum yield of photoinduced cycloaddition is very low, on the order of just a few percent.^10,35^ To the best of our knowledge, the only theoretical study to model the photochemistry of an effective NBD-QC molecular switch is that by Hernández and co-workers.^36^ These authors compared the photorelaxation dynamics of unsubstituted norbornadiene, and the norbornadiene derivative in which **R2** and **R3** are methyl groups, while **R5** and **R6** are nitrile groups.

There are ongoing efforts to optimize the performance of NBD-QC molecular switches. In particular, several studies over the past decade^15,37−46^ have explored the “push-pull” substitution pattern, in which **R2** is an aromatic electron-donating moiety, **R3** is an electron-withdrawing group, and **R5** and **R6** are hydrogen atoms. This arrangement of substituents shifts the onset of light absorption toward longer wavelengths.^37^ In some designs, a single aromatic electron-donating moiety is shared between two or three norbornadiene units, each of which can undergo photoinduced cycloaddition.^38,39,46^ This molecular architecture substantially increases the ESD relative to compounds with only one norbornadiene unit.^38,39,46^

There is only limited data on how push–pull substitution affects the photoinduced cycloaddition reaction and its quantum yield. In order to address this issue, in the present study, we used electronic structure calculations to investigate the photoisomerization mechanisms of two representative push–pull NBD-QC photoswitches. In particular, for one of the two compounds under study, we modeled the relaxation dynamics following irradiation near the origin of the lowest photoabsorption band.

The rest of this paper is organized as follows. First, we provide an outline of the simulation methodology. We then move on to discuss the key features of the photoisomerization mechanisms: the topographies of the relevant potential energy surfaces (PESs), the structures of the excited electronic states involved in photoisomerization, and the sequence of events following photoexcitation. Finally, we tie in our findings with the available data on the photochemistry of push–pull NBD-QC switches.

For the sake of brevity, in the main body of this paper we provide only a short outline of the simulation methodology. The detailed description of the computational setup, including the explanation for the choice of simulation methods and some of the adjustable parameters, is relegated to the Supporting Information (SI).

The fact that there are many unknowns regarding the photochemistry of push–pull NBD-QC switches made it necessary to perform a range of calculations aimed at elucidating different aspects of the energy capture process. In order to gain a measure of how the extension of the π-bonding system affects the photochemistry of these systems, we considered two compounds, denoted **I** and **II**, whose structures are given in Figure 1. Both compounds were designed and synthesized by Quant and co-workers,^37^ and they can be considered representative of the broader class of push–pull NBD-QC switches. Compound **I**, in which **R2** is a phenyl group and **R3** a nitrile group, is the prototypical example of a push–pull NBD-QC switch. In refs^37, 40^, it was used as a benchmark for evaluating the effect of modifying the electron-donating moiety. Compound **II**, in turn, showcases the extension of the conjugated π-bonding system with an ethynyl linker, which is a general design strategy to shift the onset of light absorption toward longer wavelengths.^37,39^

All calculations were performed in vacuum (which is to say, for isolated molecules of compounds **I** and **II**). The neglect of solvent effects is justified by the fact that our main reference point will be the study by Quant et al.,^37^ where spectroscopic measurements and the determination of quantum yields were performed in toluene, a nonpolar and weakly interacting solvent.

In the first instance, we mapped out the ground- and excited-state PESs of molecules **I** and **II**. In these so-called static calculations, we employed the mixed-reference spin-flip variant of time-dependent density functional theory^47−51^ (MRSF-TDDFT). Within the framework of MRSF-TDDFT, the target electronic states are generated through spin-flipping excitations from a specially designed reference state, which is a hypothetical “mixture” of the *M*_*S*_ = +1 and *M*_*S*_ = – 1 components of the Kohn–Sham triplet state. Singlet states, triplet states (the *M*_*S*_ = 0 components), and quintet states can be obtained in this manner. Crucially, MRSF-TDDFT is able to describe dynamical as well as static correlation effects. Unlike many of the other spin-flip methods,^52−55^ it does not suffer from significant spin contamination. These features make it well suited to the study of ground- and excited-state PESs.

The MRSF-TDDFT calculations were carried out with the program OpenQP, version 1.0.^56^ The reference state was the mixed state which consisted of the *M*_*S*_ = +1 and *M*_*S*_ = −1 components of the restricted open-shell Kohn–Sham (ROKS) triplet state. (Recently, another option has been implemented, which employs unrestricted Kohn–Sham orbitals.^57^) For these calculations, we employed the DTCAM-VAEE exchange-correlation functional^58,59^ in combination with the def2-SV(P) basis set.^60^

Photoisomerization reactions are often mediated by conical intersections (CIs) between excited electronic states and the ground state. As we will demonstrate later on, this is also the case for compounds **I** and **II**. Accordingly, we optimized minimum-energy conical intersection (MECI) geometries along the S_1_/S_0_ CI seams of these compounds. These geometry optimizations made use of the penalty function method of Ciminelli and co-workers.^61^

It is also of interest to examine the electronic structures of the low-lying excited states of the molecular switches under study. Here, in order to avoid some conceptual difficulties associated with MRSF-TDDFT and other spin-flip methods, we used instead the (spin-conserving) second-order approximate coupled cluster singles and doubles^62^ (CC2) method. (With spin-flip methods, the analysis of electronic structures is complicated by the choice of the reference state, which is an open-shell state.)

The CC2 calculations were performed in the program Turbomole, version 7.4.0.^63,64^ The electronic excitation spectra were characterized through single point calculations at ground-state equilibrium geometries optimized at the MRSF-TDDFT level. The structures of the low-lying excited states were visualized by plotting natural transition orbitals^65^ (NTOs) for the transitions from the ground state. (As an alternative way of depicting the electronic structures of these excited states, we also plotted their particle and hole densities.^66^ These plots are shown in Section S2 of the SI.) We used the aug-cc-pVDZ basis set,^67^ and we imposed the spin-component scaling (SCS) modification of CC2 theory,^68^ which improves its accuracy for excitation energies into Rydberg states.^69^ In order to emphasize the use of SCS, in what follows we will refer to this level of theory as SCS-CC2.

Another of our aims was to obtain information on the time scale and the sequence of events during the photoinduced cycloaddition reaction. To that end, we carried out nonadiabatic molecular dynamics (NAMD) simulations of the relaxation process of compound **II** following photoexcitation near the origin of the first absorption band. The dynamics of the molecule was modeled with the fewest switches surface hopping algorithm.^70−75^ In this approach, the wave packet of the system is represented as a set of mutually independent semiclassical trajectories. In each trajectory, the motions of the nuclei are described using classical mechanics, while the time-evolution of the electronic wave function is treated quantum-mechanically. We propagated *N*_trajs_ = 100 trajectories for a period of 600 fs. The simulations included states S_0_, S_1_, and S_2_.

In the course of the static calculations, we found that the reference ROKS calculation used by MRSF-TDDFT occasionally suffered from convergence issues. In order to circumvent this difficulty, for the purposes of the NAMD simulations we switched to the conventional variant of spin-flip time-dependent density functional theory^53,55^ (SF-TDDFT). In this case, the reference state is the *M*_*S*_ = +1 component of the unrestricted Kohn–Sham (UKS) triplet state, which is much easier to converge. The conventional SF-TDDFT calculations were performed in the program Q-Chem, version 5.1.2.^76,77^ We employed the 50–50 exchange-correlation functional^53^ in combination with the def2-SV(P) basis set.

The simulated dynamics was monitored with the following two parameters. The electronic state of the molecule was described in terms of the classical populations of states S_0_ to S_2_. As per the usual convention, the classical population (*P*_*j*_) of state *j* is defined as the fraction of trajectories that are currently evolving in that state:
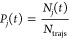
1

In order to track the progress of the photoinduced cycloaddition reaction, we defined parameter R̅ as the average of the C2–C6 and the C3–C5 distances:
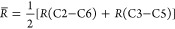
2

In each simulated trajectory, the molecule was considered to have undergone photoisomerization if R̅ decreased to less than 1.8 Å at any point during the trajectory. The quantum yield of photoisomerization was estimated as the fraction of trajectories which met this criterion.

We begin the discussion of the simulation results with the topographies of the ground- and excited-state PESs of the compounds under study. Figure 2 depicts the energy minima and S_1_/S_0_-MECI structures obtained at the MRSF-TDDFT level of theory. As expected, for both of the systems under study, we found two minima on the PES of state S_0_, which correspond to the NBD and the QC isomers. These are denoted S_0_-NBD-**I** and S_0_-QC-**I** for compound **I**, and analogously for compound **II**. Moreover, for both compounds, we detected a CI seam between states S_1_ and S_0_ that is located, informally speaking, part of the way between the NBD and the QC isomers. A comparison with the results of earlier computational studies^31−33^ indicates that this CI seam is the counterpart of the S_1_/S_0_ CI seam of unsubstituted norbornadiene. For each compound, we optimized the MECI structure along this S_1_/S_0_ CI seam (denoted S_1_/S_0_-MECI-**I** and S_1_/S_0_-MECI-**II**).

**Figure 2:**
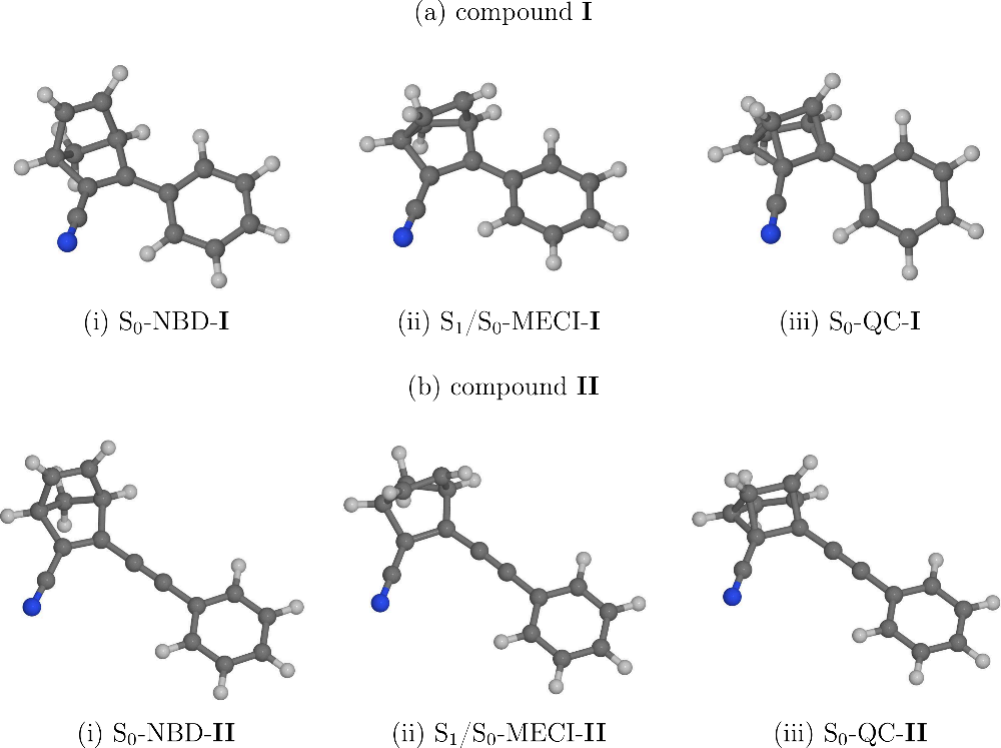
Catalog of the molecular structures of compounds **I** and **II** predicted by the MRSF-TDDFT method.

In order to determine the role of the S_1_/S_0_ CI seams in the photorelaxation processes of compounds **I** and **II**, we scanned their ground- and excited-state PESs along reaction paths corresponding to the conversion of the NBD isomer into the QC isomer through the S_1_/S_0_ MECI. The reaction paths were constructed through linear interpolation in internal coordinates (LIIC). This was done as follows. The NBD isomer, the S_1_/S_0_ MECI, and the QC isomer were described in terms of a common system of internal coordinates. The internal coordinate system was set up with the use of the subprogram “define” of Turbomole. Then, the molecular geometry was interpolated linearly in terms of these internal coordinates, starting from the minimum-energy geometry of the NBD isomer, through the S_1_/S_0_ MECI, and to the minimum-energy geometry of the QC isomer. Finally, the energies of states S_0_ to S_3_ were evaluated through single-point calculations along the resulting reaction path.

We note here that using the LIIC procedure introduces a certain element of arbitrariness. This is because an interpolated reaction path depends on the choice of the internal coordinate system. Still, in practice, LIIC tends to provide physically reasonable reaction paths. By construction, the resulting reaction paths are smooth, in the sense that the molecular geometry varies continuously (linearly) along the reaction path. The main advantage is, of course, that one avoids having to optimize the reaction path, which can be a difficult proposition. Indeed, this approach is quite popular in the computational chemistry community – for example, it has been used in such studies as refs (78−84). (This list is not exhaustive, it is merely meant as an illustration of the widespread use of this technique.) In order to demonstrate that the reaction paths that we have generated are physically reasonable, and also to enable other researchers to reproduce our results, we have included them as part of the SI.

The results of the PES scans are shown in Figure 3. The general topographies of the PESs are similar for compounds **I** and **II**. As the molecule is displaced along the reaction coordinate away from the NBD isomer toward the S_1_/S_0_ MECI structure, the energy of state S_1_ decreases steadily. In other words, the topography of the S_1_ PES favors relaxation from the Franck–Condon geometry (the ground-state equilibrium geometry of the NBD isomer) toward the S_1_/S_0_ MECI. At, or near, the MECI structure, the molecule will presumably undergo internal conversion to the singlet ground state (S_0_). Once in state S_0_, some part of the nuclear wave packet will continue its motion along the reaction coordinate toward the QC isomer. The remainder will be reflected back toward the NBD isomer.

**Figure 3:**
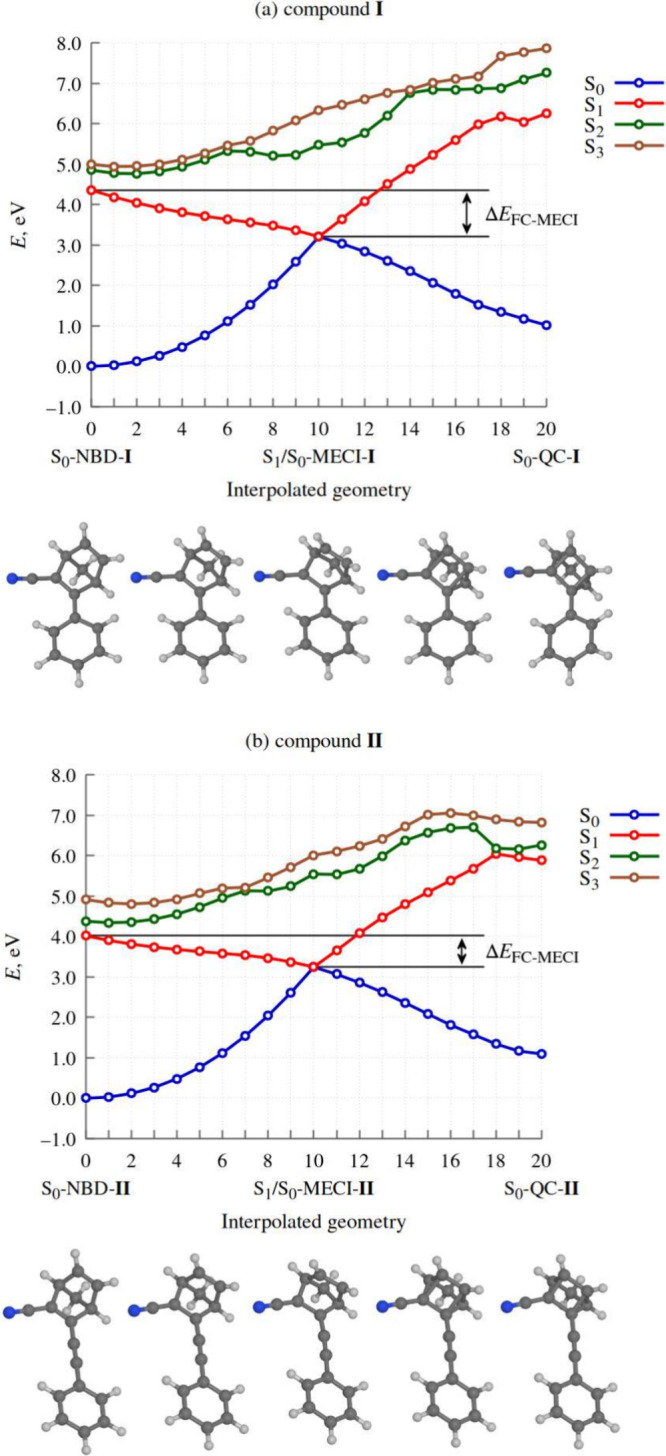
Energies of the states S_0_ to S_3_ of (a) compound **I** and (b) compound **II** along reaction paths leading from the NBD isomer, through the S_1_/S_0_ MECI structure, and to the QC isomer. The reaction paths were generated by interpolating the molecular geometry between these three structures. The molecular geometries comprising the reaction path are numbered from 0 to 20. Furthermore, the evolution of molecular geometry is shown in the insets at the bottom. The zero of the energy scale corresponds to the adiabatic energy of the NBD isomer.

As alluded to above, the driving force for relaxation to the CI seam is provided by the slope of the PES of state S_1_ in the region between the Franck–Condon geometry and the S_1_/S_0_ MECI structure. In Figure 3, the energy difference between state S_1_ at the Franck–Condon geometry and the MECI structure is labeled Δ*E*_FC-MECI_. This quantity turns out to be highly sensitive to the extension of the conjugated π-bonding system. Let us first consider unsubstituted norbornadiene as a reference point. According to the extended multistate complete active space second-order perturbation theory^85^ (XMS-CASPT2) calculations by Cooper and Kirrander,^33^ in unsubstituted norbornadiene Δ*E*_FC-MECI_ takes a value of roughly 2 eV. (The exact value depends on the choice of active space.^33^) This means that, in unsubstituted norbornadiene, the PES of state S_1_ is extremely steep. For compound **I**, the MRSF-TDDFT calculations predict a considerably lower Δ*E*_FC-MECI_ value of 1.15 eV. For compound **II**, Δ*E*_FC-MECI_ is lower still, at 0.77 eV. Thus, the increased delocalization of state S_1_ reduces, but does not eliminate, the driving force for relaxation to the CI seam. In fact, even in compound **II**, Δ*E*_FC-MECI_ remains fairly large in absolute terms.

The question then presents itself, if unsubstituted norbornadiene has a steeper S_1_ PES than either compound **I** or compound **II**, why do the latter compounds show higher quantum yields of photoinduced cycloaddition? We hypothesize that an excessively steep S_1_ PES is actually detrimental to cycloaddition. One reason why this may be the case is that, if relaxation toward the CI seam releases a large amount of energy into the vibrational modes, the molecule may encounter the CI seam at a strongly deformed geometry that is not conducive to cycloaddition. Informally speaking, the molecule might veer off the reaction path. By contrast, a more gently sloping S_1_ PES, as found in compounds **I** and **II**, will result in the molecule reaching the CI seam closer to its lowest-energy point (the MECI structure). The PES scans for compounds **I** and **II** indicate that, once the molecule is at the MECI structure, it can relax on the PES of state S_0_ to either the NBD isomer, or the QC isomer. If the molecule already has some momentum along the reaction coordinate, it will continue moving in the same direction, and reach the QC structure.

It is also of interest to inspect the branching space vectors (the gradient difference vector – GDV – and the nonadiabatic coupling vector – NACV) between the intersecting states at the S_1_/S_0_-MECI structures of compounds **I** and **II**. Accordingly, we calculated these vectors at the MRSF-TDDFT level of theory, and we show them in Figure 4. In both compounds, the branching space vectors show a similar structure, and they correspond to different deformations of the norbornadiene moiety: the GDV mainly involves the motion of atom C3 away from atom C5, and the motion of atom C6 away from atom C2. The NACV, in turn, corresponds to the elongation of the C2=C3 and the C5=C6 bonds, accompanied by the contraction of the C2–C6 and the C3–C5 distances. In qualitative terms, the NACV is related to the change in the bonding pattern on going from the NBD isomer to the QC isomer.

**Figure 4:**
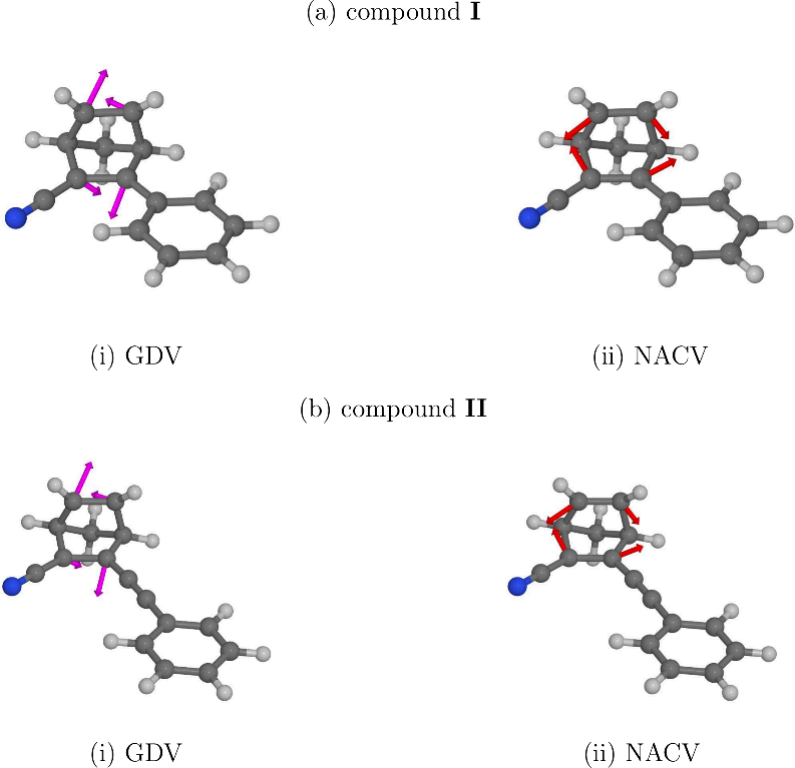
Branching space vectors of (a) compound **I** and (b) compound **II** at their respective S_1_/S_0_-MECI structures.

In the picture afforded by the fewest switches surface hopping algorithm, internal conversion is driven by the movement of nuclei parallel, or antiparallel, to the NACV between the initial and the final state. (See Section S3.1 of the SI.) Thus, the structures the S_1_–S_0_ NACVs of compounds **I** and **II** indicate that, in both compounds, internal conversion from state S_1_ to state S_0_ is directly caused by ring closing.

In closing the discussion of the PESs of compounds **I** and **II**, we note that both compounds likely possess other S_1_/S_0_ CI seams in addition to the one that is associated with photoinduced cycloaddition. For example, the presence of phenyl groups presumably gives rise to S_1_/S_0_ CIs associated with deformations of the six-membered rings.^86−88^ However, the NAMD simulations for compound **II** (which will be discussed later on) indicate that internal conversion happens exclusively at the ring-closing CI seam. Since other CI seams appear to be irrelevant to the mechanism of photoinduced cycloaddition, in this study we did not attempt to locate and characterize them.

Our next order of business will be to examine the electronic excitation spectra of the NBD isomers of compounds **I** and **II**. The vertical excitation spectra calculated at the SCS-CC2 level of theory are summarized in Table 1. For each compound, the lowest singlet excited state (S_1_) is a bright ππ*-type state. In compound **I**, this state has a vertical excitation energy of 4.609 eV, while in the case of compound **II**, the vertical excitation energy is slightly lower, at 4.376 eV. In each compound, state S_1_ has by far the largest oscillator strength from among the low-lying excited states, which means that it makes the main contribution to the lowest absorption band of the given compound.

**Table 1:** Vertical Excitation Spectra of the NBD Isomers of Compounds **I** and **II**^a^

Compound	State	Δ*E*, eV	*f*	μ, D
**I**	S_0_			4.3
	S_1_ (ππ*)	4.609	0.3117	5.3
	S_2_ (ππ*)	4.687	0.0064	4.7
	S_3_ (ππ*)	5.422	0.1068	7.9
	S_4_ (πR)	5.811	0.0044	1.9
	S_5_ (ππ*)	6.114	0.1745	3.5
	S_6_ (πR)	6.174	0.0295	2.4
**II**	S_0_			4.2
	S_1_ (ππ*)	4.376	0.6266	5.5
	S_2_ (ππ*)	4.768	0.0024	4.5
	S_3_ (ππ*)	5.075	0.0984	6.4
	S_4_ (ππ*)	5.624	0.1403	5.6
	S_5_ (πR)	5.744	0.0031	1.6
	S_6_ (ππ*)	5.976	0.0454	4.2

a Δ*E* is the vertical excitation energy, and *f* is the corresponding oscillator strength. *μ* is the (relaxed) electric dipole moment of the given state in units of debye (D). The spectra were calculated at the SCS-CC2/aug-cc-pVDZ level of theory at ground-state minimum-energy geometries optimized with the MRSF-TDDFT method.

Quant et al.^37^ measured the absorption spectra of compounds **I** and **II** in toluene solution. The lowest absorption band of compound **I** has its maximum at 309 nm, which corresponds to a photon energy of 4.01 eV. In the case of compound **II**, the band maximum is located at 331 nm, or 3.75 eV. Thus, the simulations correctly predict that the insertion of the ethynyl linker between the norbornadiene moiety and the phenyl moiety achieves a slight red shift of the lowest absorption band.

Figure 5 visualizes the electronic structures of state S_1_ of each compound by showing the dominant NTO pair for the transition from state S_0_. In both compounds, state S_1_ is delocalized over the entire π-bonding system. More insight into its electronic structure can be obtained by inspecting the bonding interactions in the hole (initial) and particle (final) orbitals. In each compound, the hole orbital has bonding character between C2 and C3, and also between C5 and C6, but antibonding between C2 and C6, and also between C3 and C5. Conversely, the particle orbital is antibonding between C2 and C3 and between C5 and C6, but bonding between C2 and C6 and between C3 and C5. In qualitative terms, states S_1_ of compounds **I** and **II** can be thought of as delocalized counterparts of state S_1_ of unsubstituted norbornadiene, in which the hole and the particle orbitals have an analogous bonding and antibonding pattern.^33^

**Figure 5:**
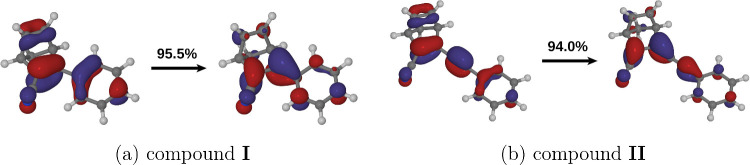
Dominant NTO pairs for the S_0_ → S_1_ transitions of (a) compound **I** and (b) compound **II**, plotted in the form of isosurfaces with isovalues of ± 0.05 *a*_0_^–3/2^. The eigenvalue (λ_*i*_) for each hole-particle NTO pair is given in terms of a percentage contribution.

The fact that cycloaddition begins in the spectroscopically bright excited state means that the choice of substituents **R2** and **R3** affects the PES of the reactive electronic state as well as the absorption spectrum of the given compound. This explains the experimental observation that the photoisomerization quantum yield and the absorption spectrum are both sensitive to even relatively minor structural modifications, such as replacing one electron-donating group with another.^37,40^

Another point of note is that state S_1_ of either compound does not possess an intramolecular charge transfer (ICT) character. This finding has implications for the design of push–pull NBD-QC photoswitches: there is apparently no need for substituent **R2** to be strongly electron-donating in character; a weakly electron-donating group, as in compounds **I** and **II**, seems adequate.

Returning now to the analysis of the vertical excitation spectrum, compounds **I** and **II** each possess a spectroscopically dark S_2_ state, which is another ππ*-type state. State S_2_ lies close enough in energy to state S_1_ that it may play a role in the photorelaxation processes of these compounds following irradiation near the origin of the lowest absorption band. This is the reason we included that state in the NAMD simulations for compound **II**.

In both of the compounds under study, S_3_ and all higher excited states are well separated in energy from the bright state S_1_. It follows that these higher excited states are not involved in these compounds’ photorelaxation dynamics. In particular, among the higher excited states, we find Rydberg (πR-type) states, which arise from the excitation of an electron from a compact π-type orbital into a diffuse (Rydberg-type) orbital.

We now move on to discuss the relaxation dynamics of the NBD isomer of compound **II** following the irradiation of its first absorption band. Figure 6 provides an overview of the results of the NAMD simulations. Accompanying this data, as part of the SI, we include animations of ten representative simulated trajectories. Each animation shows the time-evolution of the molecular geometry, and the energies and populations of states S_0_, S_1_, and S_2_ along the given trajectory. The passage of time is indicated with a vertical black line moving along the time axis. The electronic state that is occupied at the given point in time is marked with a black circle. The initial photoexcitation occurs at *t* = 0 fs.

**Figure 6:**
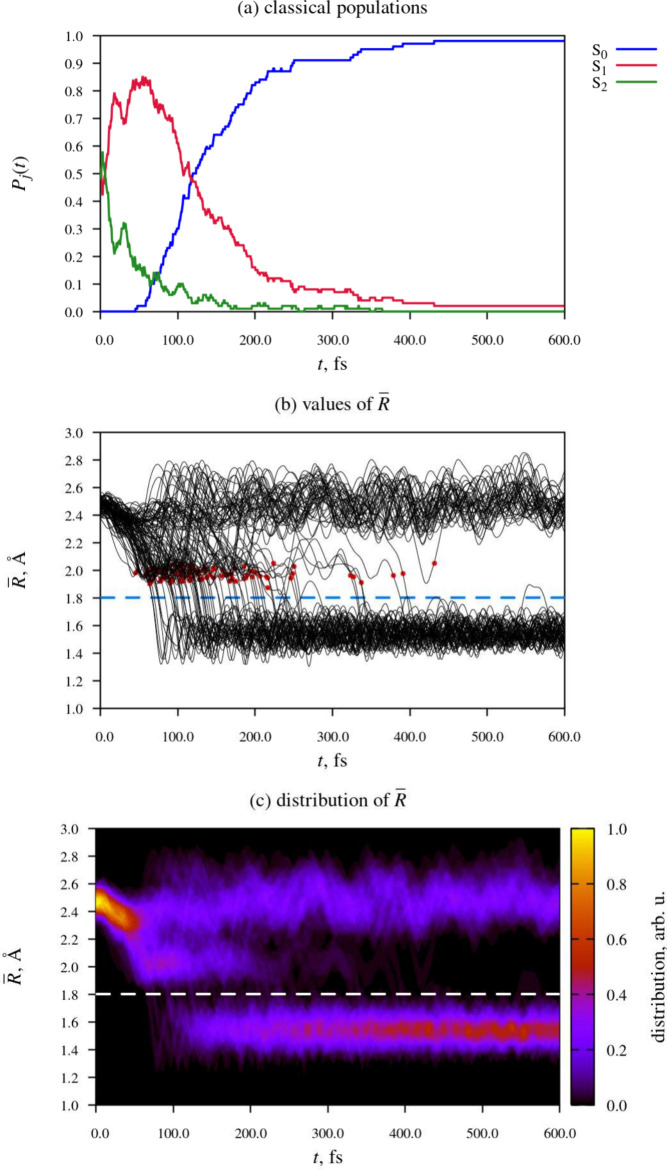
Time-evolution of electronic structure (panel (a)) and molecular geometry (panels (b) and (c)) during the relaxation dynamics of the NBD isomer of compound **II**. In panels (b) and (c), the horizontal dashed line indicates an R̅ value of 1.8 Å, which we consider as the threshold for ring closing. In panel (b), the point when the given trajectory hops into state S_0_ for the first time is marked with a red dot.

We focus first on the classical populations of the states included in the simulation, which are shown in Figure 6 (a). The slightly stepped appearance of the plot is due to the fact that, whenever a simulated trajectory hops from one state to another, the classical populations change discontinuously, as per eq 1.

At the outset of the simulations, the classical populations of states S_1_ and S_2_ were each equal to 0.50. NB: the fact that the initial classical populations of the two states were exactly equal to one another is partially a coincidence. As explained in the SI, the initial conditions for the NAMD simulations were generated stochastically in such a way as to select states S_1_ and S_2_ with probability proportional to the oscillator strength. Thereby, states S_1_ and S_2_ each just happened to be selected 50 times out of 100.

In the Franck–Condon region, states S_1_ and S_2_ are close in energy, and show strong nonadiabatic coupling. As a consequence, during the initial, roughly 50 fs-long period of time immediately following photoexcitation, many among the simulated trajectories hopped between the two states. The downward S_2_ → S_1_ hops were predominant, such that the net effect was population transfer from state S_2_ into state S_1_. Between around *t* = 20 fs and *t* = 100 fs, the majority of simulated trajectories were occupying state S_1_.

From around *t* = 50 fs, the simulated trajectories began to encounter the S_1_/S_0_ CI seam, and to hop down from state S_1_ into state S_0_. The onset of internal conversion into the singlet ground state is reflected by the steady increase in the classical population of state S_0_ starting at around *t* = 50 fs. At the end of the simulations (*t* = 600 fs), all but two among the simulated trajectories were occupying state S_0_.

Figure 6 (b) shows values of parameter R̅ in the ensemble of simulated trajectories. As an alternative way of presenting the same data, panel (c) shows the distribution of R̅ as a function of time. At *t* = 0, R̅ was narrowly distributed in the range of around 2.4 Å to 2.6 Å. The spread in the values of R̅ at the time of the initial photoexcitation reflects the spatial distribution of the nuclear wave function of the molecule in the electronic ground state.

During the first tens of femtoseconds following photoexcitation, the distribution of R̅ shifted toward lower values, while remaining fairly narrow. At around *t* = 50 fs, it began to broaden out. Between around *t* = 50 fs and *t* = 200 fs, there was a buildup of trajectories in the R̅ range of roughly 1.9 Å to 2.2 Å, in the vicinity of the MECI structure. We hypothesize that this build-up occurs because the norbornadiene moiety is fairly rigid. Presumably, for R̅ to decrease below around 1.9 Å, a number of bond lengths and angles must adjust to the new, QC-like geometry, and this causes a delay on the order of a few tens of femtoseconds.

It is informative to examine the range of molecular geometries where internal conversion to the ground state takes place. In Figure 6, the point when a given trajectory hops into state S_0_ for the first time is marked with a red dot. It can be seen that the nonadiabatic transitions take place over a narrow range of R̅ from around 1.9 Å to 2.1 Å. This indicates that internal conversion is mediated by only one S_1_/S_0_ CI seam – the one that is associated with the MECI structure identified in the static calculations.

The earliest instance of ring closing (in the sense that R̅ decreased to below the threshold of 1.8 Å) occurred at *t* = 67 fs. The median time of ring closing was 138 fs, and the mean was 164 fs. This result places the photoinduced cycloaddition reaction of compound **II** among the fastest known photoisomerization reactions. Its short time scale is presumably due to a combination of factors. First, in the NBD isomer, the C2**=**C3 and the C5**=**C6 bonds are positioned fairly close to each other. Second, the topography of the PES of state S_1_ favors relaxation from the Franck–Condon region toward the S_1_/S_0_ CI seam mediating cycloaddition. Lastly, the – C5H**=**C6H– fragment is relatively light, which allows it to move fast.

Those trajectories which did not undergo cycloaddition were reflected back toward the NBD structure. In Figure 6 (b) and (c), these trajectories appear from around *t* = 100 fs in the R̅ range of around 2.3 Å to around 2.7 Å. The reason this range is fairly broad is that the molecule was in a highly excited vibrational state (the so-called “hot” electronic ground state), and the heavy-atom skeleton of the norbornadiene moiety was undergoing large-amplitude vibrations.

The quantum yield estimated on the basis of the simulation results is 0.60 ± 0.10, somewhat higher than the experimental value of 0.39 reported in ref (37). Here, the uncertainty in simulation-based value is the binomial confidence interval at a 95% confidence level. It represents the statistical uncertainty arising from the finite number of simulated trajectories; it does not take into account other sources of error, such as the approximations inherent in the NAMD method. The experimental value pertains to compound **II** in toluene solution, photoexcited at 310 nm.^37^

The overestimation of the quantum yield may be partially due to the relatively short time frame of the simulations. The QC isomer is formed in the “hot” ground state. Some of the QC molecules may subsequently revert to the NBD isomer through a thermal cycloreversion reaction before they are able to dissipate heat into the surrounding solvent. This effect is not accounted for by the present simulations, first because the solvent is not included, and second because the simulation time is shorter than the time scale of vibrational cooling of molecules in hot ground states, which is on the order of picoseconds.^89−91^ Other possible reasons for the overestimation of the quantum yield are the various approximations inherent in the simulations, such as the fact that the PESs are being calculated at the SF-TDDFT level of theory. Still, the discrepancy between simulation and experiment is, we believe, small enough that it does not cast doubt on the qualitative accuracy of the simulations.

A more direct comparison of the simulation results to experimental data is complicated by the fact that the NBD → QC photoisomerization reactions of compounds **I** and **II** have not been characterized with the use of time-resolved spectroscopic methods, such as transient absorption (TA) spectroscopy. To the best of our knowledge, the only study to date to have investigated the photochemistry of a push–pull NBD derivative with time-resolved methods was the work by Alex and co-workers.^92^ These authors used TA spectroscopy to follow the photorelaxation processes of the NBD and the QC isomers of the photoswitch in which **R2** is a 4-(*N*,*N*-dimethylamino)phenyl group (*p*-C_6_H_4_–N(CH_3_)_2_), **R3** is a methyl carboxylate group (CO_2_–CH_3_), and **R5** and **R6** are hydrogen atoms.

However, the relaxation mechanism of the NBD isomer of that compound is substantially different from what our present simulations predict for compounds **I** and **II**. More specifically, in NBD isomer of the compound studied by Alex et al., the lowest singlet excited state has substantial ICT character.^92^ This is attributable to strong electron-donating ability of the 4-(*N*,*N*-dimethylamino)phenyl group. Following the irradiation of the lowest photoabsorption band of the compound studied by Alex et al. in acetonitrile solution, the ICT state shows a relatively long lifetime of ca. 350 ps.^92^

The much shorter excited-state lifetime of the NBD isomer of compound **II** likely contributes to the photostability of that compound – that is to say, its resistance to damage brought about by the action of light (photodegradation). Regardless of whether a photoexcited molecule of the NBD isomer undergoes isomerization into the QC form, it rapidly returns to the electronic ground state, after which it will dissipate excess energy in the form of heat. This reduces the amount of time when it is susceptible to damage, such as by an irreversible photoisomerization reaction,^92^ or oligomerization.^93,94^

In summary, in this study, we employed electronic structure calculations to model the energy capture processes of two representative push–pull NBD-QC photoswitches at the single-molecule level. The simulation results indicate that the photoinduced cycloaddition reactions of these compounds take place in their respective S_1_ states, which are also responsible for light absorption. The topography of the PES of state S_1_ creates a strong driving force for relaxation toward the norbornadiene-like CI seam, which mediates internal conversion to the ground state. Ring closing is predicted to take place on an extremely short time scale on the order of 150 fs. This is likely to be a factor behind the high photostability of push–pull NBD-QC switches.

Interestingly, and somewhat surprisingly, the calculations also show that the push–pull NBD-QC switches have more mildly sloping S_1_ PESs than the parent compound – unsubstituted norbornadiene. Since these compounds achieve significantly higher photoisomerization quantum yields than unsubstituted norbornadiene, it seems that a gentle slope of the S_1_ PES is more favorable to ring closing than a steep slope. We hypothesize that this is because a gently sloping S_1_ PES causes the molecule to encounter the S_1_/S_0_ CI seam close to its lowest-energy point.

On the methodological side, an important finding by our work is that spin-flip methods such as MRSF-TDDFT are able to describe the ground- and excited-state PES of push–pull NBD-QC switches along the reaction coordinate for photoisomerization. Owing to the fact that MRSF-TDDFT can be employed as a “black box” method, in the sense that it does not require the user to select an active space or other simulation parameters that are specific to a given molecule, it potentially lends itself to the systematic study of structure–property relationships in compounds of this type.

## Data Availability

In the interest of scientific reproducibility, the simulated NAMD trajectories have been made available for download at the Zenodo repository: 10.5281/zenodo.14881707. The NAMD simulations were performed with the use of a “wrapper” program, which acted as an interface to Q-Chem. Its source code is available for download from the Zenodo repository: 10.5281/zenodo.14910302. Researchers interested in using this program are also encouraged to contact the authors directly.

## Supporting Information Available

Detailed description of computational methodology (PDF)
Animations of simulated trajectories (ZIP)
Animations of simulated trajectories (ZIP)
Selected input and output files from the static calculations. Interpolated reaction paths for the photoisomerization of compounds **I** and **II** (ZIP)
Transparent Peer Review report available (PDF)
