# Supplementary material for: Simulating the Energy Capture Process in Push–Pull Norbornadiene-Quadricyclane Photoswitches
Source: J Phys Chem Lett. 2025 Apr 23;16(17):4315–25. doi: 10.1021/acs.jpclett.5c00634 (PMC12051204; doi:10.1021/acs.jpclett.5c00634)
Supplement: Supplementary file 1 — jz5c00634_si_001.pdf [file jz5c00634_si_001.pdf]

Supporting Information for

Simulating the Energy Capture Process in Push-Pull Norbornadiene-Quadricyclane Photoswitches

Michał Andrzej Kochman[†‡], Bo Durbeej[¶]

[†] Institute of Physical Chemistry, Polish Academy of Sciences, Ul. Marcina Kasprzaka 44/52, 01-224 Warsaw, Poland
[‡] Theoretical Chemistry, Ruhr University Bochum, Universitätsstraße 150, 44801 Bochum, Germany
[¶] Division of Theoretical Chemistry, Department of Physics, Chemistry and Biology (IFM), Linköping University, 58183 Linköping, Sweden

e-mail: mkochman@ichf.edu.pl, bodur@ifm.liu.se

Contents

S1 Static Calculations **2**
S1.1 Computational Parameters 2
S1.2 Benchmark Calculations 4

S2 Calculation of Electronic Excitation Spectra **9**

S3 NAMD Simulations **11**
S3.1 Fewest Switches Surface Hopping 11
S3.2 Electronic Structure Calculations 14
S3.3 Initial Conditions 16
S3.4 Analysis of UKS Reference State 17

References **19**

S1 Static Calculations

This section expands on the main body of our paper by providing more details on the static calculations. This series of calculations consisted of geometry optimizations and potential energy surface (PES) scans for compounds **I** and **II**. Our electronic structure method of choice was the mixed-reference spin-flip variant of time-dependent density functional theory[1–5] (MRSF-TDDFT). MRSF-TDDFT belongs to the spin-flip class of electronic structure methods,[6–11] which are based on the idea that the target states (i.e., the electronic states of interest) are generated through spin-flipping excitations from a high-spin reference state. The target states may, in general, include the ground state of the system as well as some of its excited states. This enables spin-flip methods to describe some static correlation effects in the target states, including in the ground state.

In the case of MRSF-TDDFT, the reference state is a hypothetical "mixture" of the two high-spin components ($M_S = +1$ and $M_S = -1$) of the Kohn–Sham triplet state. The target states are obtained via the linear response formalism. Singlet states, triplet states (the $M_S = 0$ components), and quintet states can be obtained in this manner. Owing to the specially designed mixed reference state, MRSF-TDDFT almost completely eliminates spin contamination in the target states. This represents a major improvement over the conventional variant of spin-flip time-dependent density functional theory (SF-TDDFT), which gives rise to varying degrees of spin contamination.[7,11]

While the available data on the performance of MRSF-TDDFT in the calculation of PESs is very encouraging,[12,13] it is still a relatively new and untested method. To the best of our knowledge, it has not previously been applied to study $[2+2]$ cycloaddition reactions (not even for a model system such as unsubstituted norbornadiene). For this reason, we could not automatically assume that it gives a satisfactorily realistic description of the relevant PESs of NBD-QC molecular switches. In order to gain a measure of the accuracy of MRSF-TDDFT for compounds of this class, we carried out test calculations in which we compared PESs predicted by that method to the benchmark provided by extended multi-state complete active space second-order perturbation theory[14] (XMS-CASPT2). These calculations are discussed in Section S1.2 of this document.

S1.1 Computational Parameters

The MRSF-TDDFT calculations were performed in the program OpenQP, version 1.0.[15] The reference state comprised the $M_S = +1$ and $M_S = -1$ components of the restricted open-shell Kohn–Sham (ROKS) triplet state. (Recently, another option has been implemented, which employs unrestricted Kohn–Sham orbitals.[16]) We employed the DTCAM-VAEE exchange-correlation functional,[17] which is one of the doubly tuned Coulomb-attenuated method (DTCAM) series of functionals[18] optimized specifically for use with the MRSF-TDDFT method. We employed the def2-SV(P) basis set.[19] The use of this small basis set was necessitated by the fact that larger basis sets led to convergence problems in the reference ROKS calculations. The SCF convergence threshold was set to 10^{-7} E_h (hartree). Furthermore, we used the Murray-Handy-Laming[20] (MHL) radial grid with 96 radial points and 302 angular points, without pruning.

In these and all subsequent calculations, we only took into account the low-lying singlet states; we did not consider intersystem crossing (ISC) into the triplet manifold. This is justified by the fact that the simulations predict that compounds **I** and **II** have short excited-state lifetimes, such that ISC is expected to be relatively minor in importance.

In optimizations of minimum-energy conical intersection (MECI) geometries, we used the penalty function method of Ciminelli and co-workers.[21] This approach transforms the problem of optimizing the MECI into the problem of optimizing the penalty function of the following form:

$$f(\mathbf{R}) = \frac{E_1(\mathbf{R}) + E_0(\mathbf{R})}{2} + c_1 {c_2}^2 \ln \left[1 + \left(\frac{E_1(\mathbf{R}) - E_0(\mathbf{R})}{c_2} \right)^2 \right] \tag{1}$$

where $\mathbf{R}$ denotes the molecular geometry, and $E_0(\mathbf{R})$ and $E_1(\mathbf{R})$ are the energies of the intersecting states. The role of the first term of the penalty function is to ensure that the optimization lowers the average energy of the intersecting states. The second term introduces a penalty for a non-zero value of the energy gap between the intersecting states. In practice, the minimum of the penalty function coincides closely (but not exactly) with the true MECI.[22] At the minimum of the penalty function, the energy gap is small, but not exactly zero.[22] The parameters c_1 and c_2 were set to the values recommended by Ciminelli et al:[21] $c_1 = 5$ (kcal/mol)$^{-1}$ and $c_2 = 5$ kcal/mol.

On the technical side, all geometry optimizations (both of energy minima and of S_1/S_0-MECI structures) were carried out by interfacing OpenQP to the program Gaussian 09, Revision D.01.[23] In this setup, Gaussian handles the geometry optimization by calling OpenQP for the calculation of the energy and gradient. The communication between the two programs is managed by a simple C++ script. As per the default settings in Gaussian, the geometries were optimized with the use of the Berny algorithm in redundant internal coordinates.[24–31] Each optimized geometry was confirmed to be a minimum on the PES of the given electronic state through a numerical calculation of vibrational frequencies.

In the case of S_1/S_0-MECI optimizations, the script that acted as the interface between OpenQP and Gaussian calculated the value of the penalty function and its gradient, and passed on that information to Gaussian. Furthermore, we verified that the optimized MECI geometries corresponded to minima of the penalty function by calculating the Hessian of the penalty function numerically.

The S_1/S_0-MECI structures of compounds **I** and **II** were further characterized by plotting the branching space vectors – the gradient difference vector (GDV) and the nonadiabatic coupling vector (NACV) – between the intersecting states. As will be discussed in Section S3.1 in this document, internal conversion (population transfer between electronic states of the same multiplicity) is caused by the motion of nuclei parallel, or antiparallel, to the NACV between the initial and the final states. We took advantage of this property in order to identify the nuclear motions that bring about the $S_1 \rightarrow S_0$ internal conversion processes of compounds **I** and **II**.

The S_1-S_0 GDVs and NACVs of compounds **I** and **II** were calculated at their respective S_1/S_0 MECI geometries. For either compound, the GDV was calculated analytically as $\nabla E(S_1) - \nabla E(S_0)$. Because the analytical calculation of the NACV is not available at the

MRSF-TDDFT level, the calculations of the NACVs with that method were performed using the finite differences technique. Following the recommendation made in the OpenQP documentation,[32] the nuclear displacement for the purpose of calculating the NACV was set to 1×10^{-4} Å.

Note that in the NAMD simulations, we used the conventional variant of the SF-TDDFT method, and not the mixed-reference variant. In the course of the NAMD simulations, the NACVs were calculated analytically. See Section S3.2 in this document for more details.

S1.2 Benchmark Calculations

As noted in Section S1.1 above, we assessed the accuracy of MRSF-TDDFT against the benchmark provided by XMS-CASPT2. For reasons of computational tractability, we only performed benchmark XMS-CASPT2 calculations for molecule **I**, the smaller of the two molecules under study. Furthermore, we restricted ourselves to single-point calculations along the reaction path which we had constructed on the basis of MRSF-TDDFT geometry optimizations (see Figure 3 in the main body of our paper).

The XMS-CASPT2 calculations were performed in the program BAGEL.[33] We used the cc-pVDZ orbital basis set,[34] and the cc-pVTZ density fitting basis set.[35]

The orbitals to be included in the reference complete active space self-consistent field[36] (CASSCF) calculation were selected at the geometry of the NBD isomer. After some experimentation, we finally decided to include all 4 π/π^* orbitals of the nitrile group, all 4 π/π^* orbitals of norbornadiene moiety, and also 4 π/π^* orbitals of the phenyl group. Thus, the resulting active space consisted of 12 electrons distributed in 12 orbitals, which are shown in Figure S1. Furthermore, we imposed state averaging over the five lowest singlet states (i.e., S_0 to S_4) with equal weights.

At the stage of the XMS-CASPT2 calculation, we used the single state-single reference (SS-SR) contraction scheme.[37] Moreover, we imposed a vertical shift of 0.5 E_{h}.

The PESs predicted by MRSF-TDDFT and XMS-CASPT2 are compared in Figure S2. For reference, the molecular geometries comprising the reaction path are shown in Figure S3.

States S_0 and S_1 are by far the most important to us, as they are the ones directly involved in the mechanism of photoinduced cycloaddition. For these two states, MRSF-TDDFT and XMS-CASPT2 are in satisfactorily good agreement along the entire reaction path. At the S_1/S_0-MECI geometry, which was optimized with the MRSF-TDDFT method, the S_1–S_0 energy gap predicted by XMS-CASPT2 is only 0.15 eV. The small energy gap indicates that the two methods agree closely (though not exactly) on the location of the S_1/S_0 CI seam.

For states S_2, S_3, and S_4, the situation is more complex. These states are narrowly spaced in energy, and they apparently interact with one another at a number of points along the reaction coordinate. This makes it difficult to assess the degree of agreement between MRSF-TDDFT and XMS-CASPT2. For the NBD isomer (geometry 0 in Figures S2 and S3), XMS-CASPT2 predicts a significantly larger $S_0 \rightarrow S_4$ vertical excitation energy than does MRSF-TDDFT. We have no definitive explanation for this result.

On a more positive note, MRSF-TDDFT qualitatively reproduces the behavior of the S_1–S_2 energy gap that is predicted by XMS-CASPT2. Namely, at the ground-state equilibrium geometry of the NBD isomer (geometry 0), the energy gap between the two states is small

Figure S1: CASSCF active space natural orbitals of molecule **I**, depicted in the form of isosurfaces with isovalues of $\pm 0.05\ a_0^{-3/2}$. The orbitals were plotted at ground-state equilibrium geometry of the NBD isomer as optimized at the MRSF-TDDFT level.

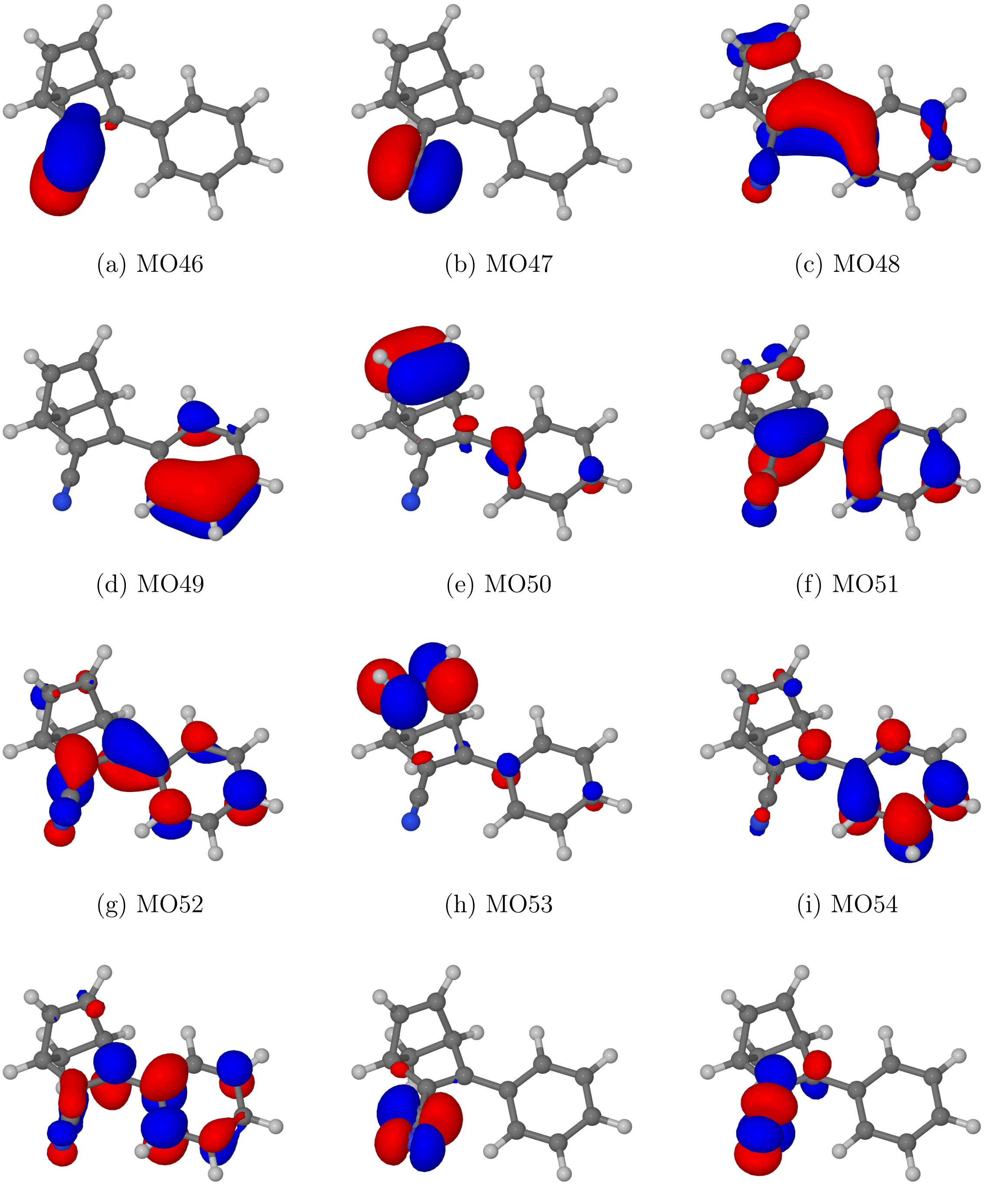

(a) MO46 (b) MO47 (c) MO48

(d) MO49 (e) MO50 (f) MO51

(g) MO52 (h) MO53 (i) MO54

(j) MO55 (k) MO56 (l) MO57

(0.62 eV at the XMS-CASPT2 level, and 0.49 eV at the MRSF-TDDFT level). It increases markedly as the molecule is displaced towards the S_1/S_0-MECI structure, and remains fairly large until the molecule is close to the ground-state equilibrium geometry of the QC isomer (geometry 20). This observation suggests that MRSF-TDDFT will not artificially overestimate the role of S_2 and the higher excited states in the reaction mechanism. This is a key finding that is no less relevant than the fact that MRSF-TDDFT predicts accurate PESs for states S_0 and S_1.

In summary, MRSF-TDDFT provides an accurate picture of the PESs of states S_0 and S_1 of compound **I**. As such, it can be safely applied to model the photoisomerization reaction of that compound. This conclusion can be extrapolated to the larger molecule **II**, in which state S_1 has a similar electronic structure as in molecule **I**. For the purposes of the present study, the fact that MRSF-TDDFT is not necessarily reliable for the higher excited states does not represent a problem, as these states do not play a significant role in the mechanisms of the NBD$\rightarrow$QC photoisomerization reactions of the two compounds under study.

Figure S2: Energies of states S_0 to S_4 of compound **I** along the reaction path for photoisomerization, calculated with (a) MRSF-TDDFT and (b) XMS-CASPT2. For ease of comparison, in panel (c), the potential energy curves obtained with the two methods are overlaid on one another. The reaction path was generated on the basis of MRSF-TDDFT geometry optimizations, and it leads from the NBD isomer, through the S_1/S_0-MECI, and to the QC isomer.

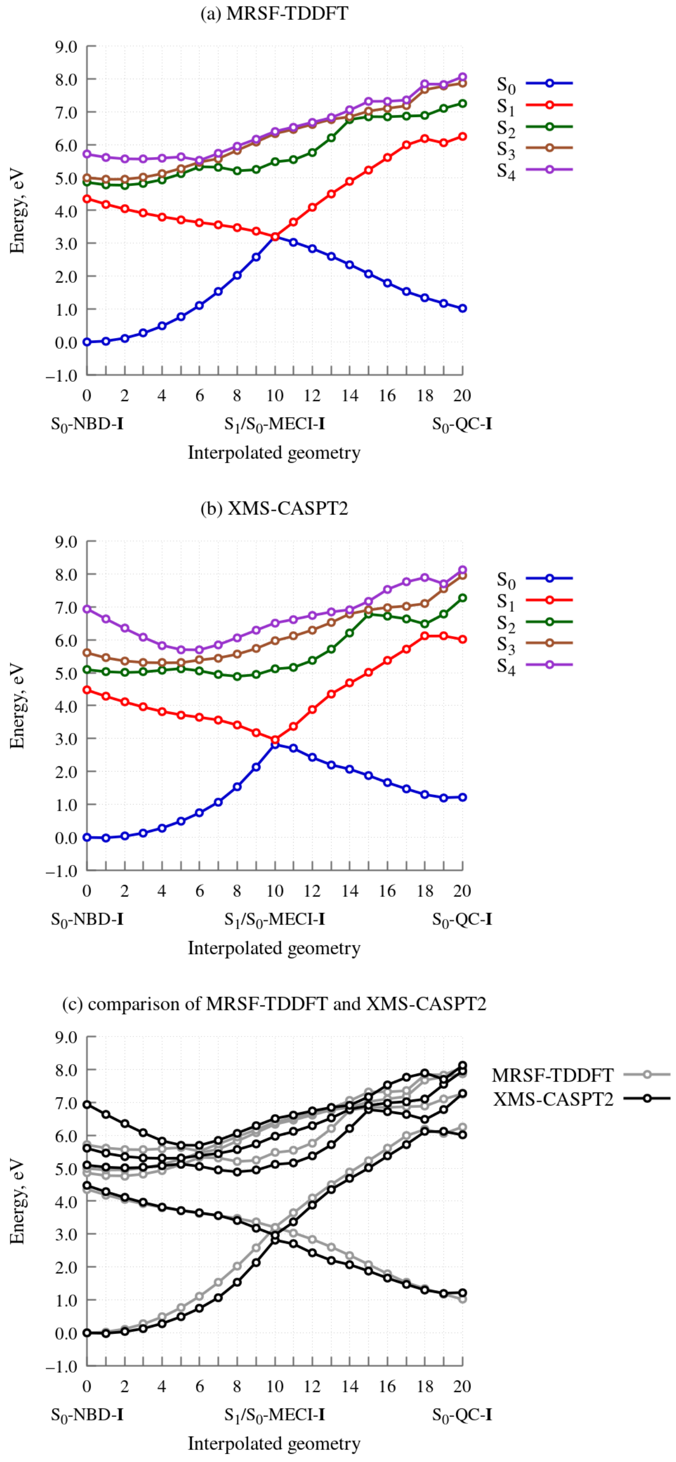

Figure S3: Molecular geometries of compound **I** along the interpolated reaction path. The geometries comprising the reaction path are numbered from 0 to 20. For the sake of clarity, only every other geometry is shown. Geometry 0 is the ground-state equilibrium geometry of the NBD isomer. Geometry 10 is the S_1/S_0-MECI structure. Geometry 20 is the ground-state equilibrium geometry of the QC isomer. The reaction path was generated by linear interpolation between these three structures in terms of internal coordinates.

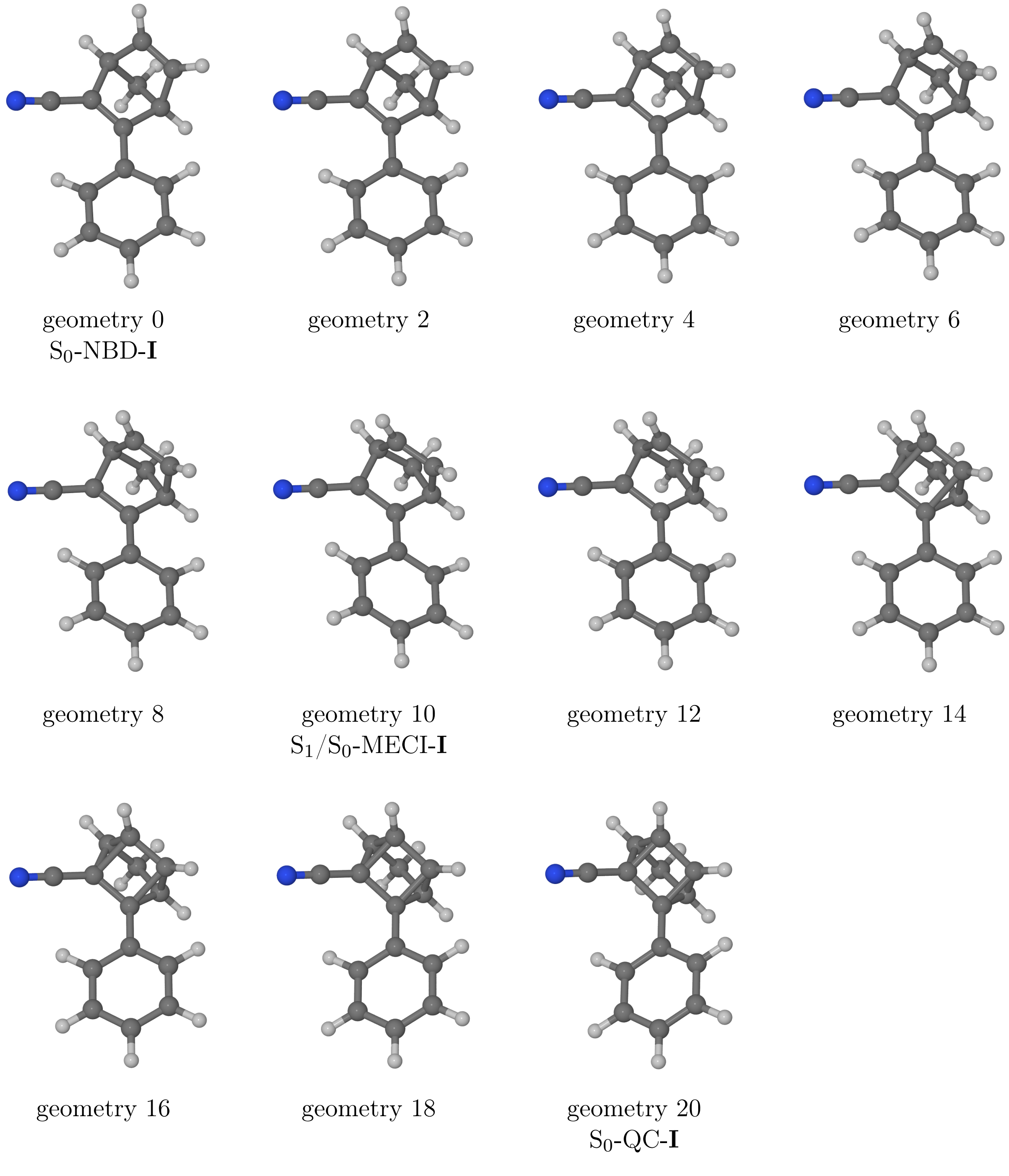

S2 Calculation of Electronic Excitation Spectra

The electronic excitation spectra of the NBD isomers of compounds **I** and **II** were calculated with the use of the second-order approximate coupled cluster singles and doubles[38] (CC2) method. We imposed the spin-component scaling (SCS) modification of CC2 theory,[39] which is expected to improve the accuracy of the calculations for excitation energies into Rydberg states.[40] The scaling factors were set to the values proposed in Ref. 41 for use with second-order Møller-Plesset perturbation theory (MP2) theory: $c_{\mathrm{SS}} = 1/3$ for the same-spin contributions to the correlation energy, and $c_{\mathrm{OS}} = 6/5$ for the opposite-spin contributions.

The SCS-CC2 calculations were performed in the program Turbomole, version 7.4.0.[42,43] In order to avoid having to re-optimize molecular geometries, we calculated the vertical excitation spectra at the ground-state equilibrium geometries optimized at the MRSF-TDDFT level. The reference state was the restricted Hartree–Fock (RHF) state. The calculations made use of the frozen core and the resolution of the identity[44–47] approximations. Transition moments were calculated with the method introduced in Ref. 48. The aug-cc-pVDZ basis set[49] was employed in combination with the default auxiliary basis set.[50]

For either compound, the electronic structure of the lowest singlet excited state was visualized by plotting the most important natural transition orbital[51] (NTO) pair for the $S_0 \rightarrow S_1$ transition. These plots can be found in Figure 4 in the main body of our paper. As an alternative way of analyzing the electronic structures of states S_1 of the two compounds, we have also generated plots of the hole and the particle densities[52] for their respective $S_0 \rightarrow S_1$ transitions. The hole and particle densities and the NTOs are related through the following expressions:[52]

$$\text{hole density:} \qquad \rho_{\mathrm{H}}(\mathbf{r}) = \sum_i \lambda_i \left(\psi_i^{\mathrm{H}}(\mathbf{r}) \right)^2 \tag{2}$$

$$\text{particle density:} \quad \rho_{\mathrm{P}}(\mathbf{r}) = \sum_i \lambda_i \left(\psi_i^{\mathrm{P}}(\mathbf{r}) \right)^2 \tag{3}$$

Here, $\psi_i^{\mathrm{H}}(\mathbf{r})$ and $\psi_i^{\mathrm{P}}(\mathbf{r})$ are, respectively, the i-th hole NTO and the i-th particle NTO associated with the $S_0 \rightarrow S_1$ transition, and λ_i is the eigenvalue corresponding to that NTO pair. For pragmatic reasons, when calculating the hole and the particle densities, we truncated the sums in equations 2 and 3 after the last term with $\lambda_i > 0.005$.

The resulting hole and particle densities are plotted in Figure S4. It can be seen that, in compound **I**, the hole density is mainly localized in the π-bonding regions of the C2=C3 bond and, to a lesser extent, on the C5=C6 bond and on the phenyl and nitrile groups. Meanwhile, the particle density is predominantly localized in the π-bonding regions of the C2–Ph and the C3–CN bonds.

In compound **II**, in turn, the hole and the particle densities are partially delocalized onto the ethynyl (C≡C) bridge connecting atom C2 and the phenyl group.

Importantly, the topographies of the hole and the particle densities confirm that the lowest singlet states of compounds **I** and **II** do not show an appreciable intramolecular charge transfer character. Indeed, in both compounds, the particle density is largely localized in the same volume of space as the hole density. This is the hallmark of a locally excited state.

Figure S4: Hole and particle densities for the $S_0 \rightarrow S_1$ transitions of (a) compound **I** and (b) compound **II.** The densities are plotted in the form of isosurfaces with isovalues of $\pm 0.005\ {a_0}^{-3}$. The numbering of atoms in the norbornadiene moiety is marked in black.

(a) compound **I**

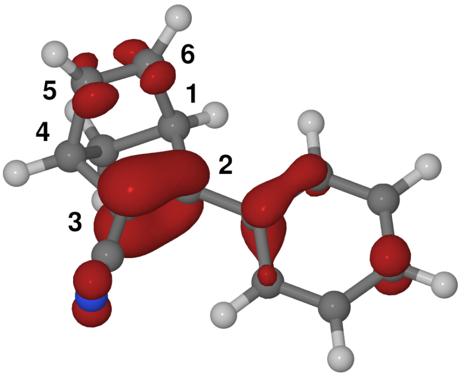

(i) hole density

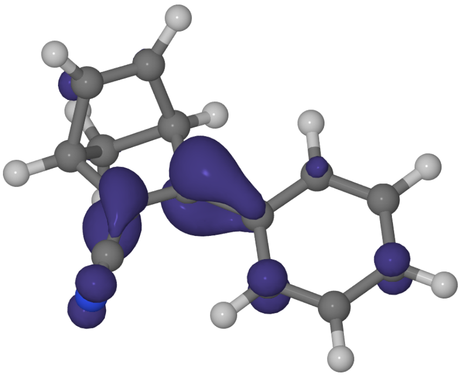

(ii) particle density

(b) compound **II**

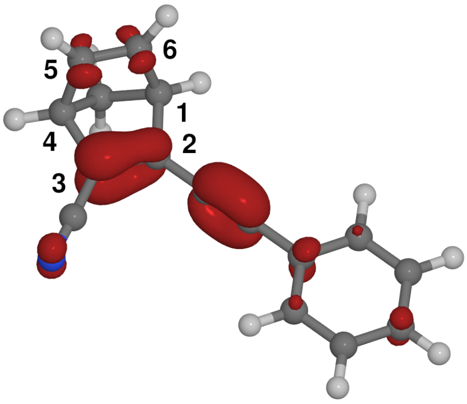

(i) hole density

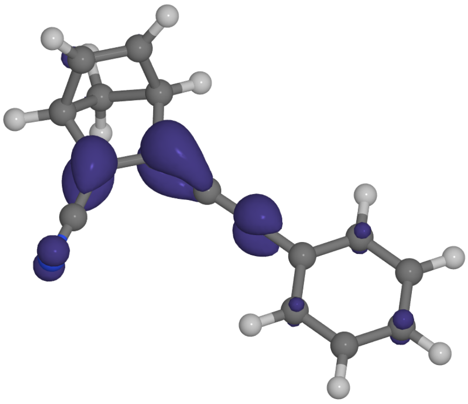

(ii) particle density

S3 NAMD Simulations

Besides the static calculations, the other major part of our study was to model the excited-state relaxation dynamics of a representative NBD-QC molecular switch. This was done for a number of reasons. Dynamical (time-resolved) simulations provide information on the timescale and the sequence of events during the relaxation process. This data may be useful for future spectroscopic studies. The reaction timescale also has implications for the photostability of the molecular switch. Moreover, simulations of this type can serve as the basis for the calculation of the photoisomerization quantum yield, which can be compared to available experimental data, and provide an indication of the realism of the simulations.

Our method of choice was the fewest switches surface hopping[53–58] variant of the nonadiabatic molecular dynamics (NAMD) method. For reasons of economy, we only performed NAMD simulations for compound **II**, whose physicochemical properties (documented in Ref. 59) are somewhat better than those of compound **I**. The simulation details are described over the following pages.

S3.1 Fewest Switches Surface Hopping

The NAMD method is a generalization of the Born-Oppenheimer molecular dynamics[60] method to the simulation of electronically nonadiabatic processes, which is to say, processes that involve a nonradiative change in the electronic state of the system. Within the framework of NAMD, the nuclear wavepacket of the system is represented as a set of mutually independent semiclassical trajectories, which is denoted $\{\mathbf{R}_i(t)\}$. In each such trajectory, the dynamics of the nuclei is described by means of classical mechanics, while the electronic structure of the molecule and its time-evolution are treated quantum-mechanically.

The electronic wave function $\Psi(\mathbf{r}, t; \mathbf{R}_i)$ along a given nuclear trajectory $\mathbf{R}_i = \mathbf{R}_i(t)$ is expressed as a linear combination of adiabatic electronic states $\{\psi_j(\mathbf{r}; \mathbf{R}_i)\}$ with time-dependent coefficients $\{a_j(t)\}$:

$$\Psi(\mathbf{r}, t; \mathbf{R}_i) = \sum_j a_j(t)\, \psi_j(\mathbf{r}; \mathbf{R}_i) \tag{4}$$

The quantity $|a_j(t)|^2$ is interpreted as the population of state j at time t along trajectory $\mathbf{R}_i$. The requirement that the electronic wave function $\Psi(\mathbf{r}, t; \mathbf{R}_i)$ is to satisfy the time-dependent electronic Schrödinger equation leads to the following system of coupled differential equations for the time-evolution of the expansion coefficients:

$$\mathrm{i}\,\hbar\,\dot{a}_k = \sum_j a_j(t) \Big(\delta_{kj}\, E_k(\mathbf{R}_i) - \mathrm{i}\,\hbar\,\dot{\mathbf{R}}_i \cdot \mathbf{d}_{kj}(\mathbf{R}_i) \Big) \tag{5}$$

where δ_{kj} denotes the Kronecker delta, $E_k(\mathbf{R})$ is the PES of the k-th adiabatic state, and $\mathbf{d}_{kj}(\mathbf{R})$ is the NACV between states k and j:

$$\mathbf{d}_{kj}(\mathbf{R}) = \langle\, \psi_k(\mathbf{r}; \mathbf{R})\, |\, \nabla_{\mathbf{R}}\, |\, \psi_j(\mathbf{r}; \mathbf{R})\, \rangle \tag{6}$$

A consequence of equation 5 is that internal conversion is driven by the motion of the nuclei parallel, or antiparallel, to the NACV between the initial and the final states. (For population transfer to take place between states k and j, the term $\dot{\mathbf{R}}_i \cdot \mathbf{d}_{kj}(\mathbf{R}_i)$ must be non-zero.) Thus, the calculation of the NACV provides a means of identifying the nuclear motions that are responsible for internal conversion.

In the fewest switches surface hopping scheme, one of the adiabatic states included in the linear expansion 4 is singled out as the occupied, or current, state in the given trajectory. The dynamics of the nuclei is propagated according to the classical equations of motion on the PES of that state:

$$\ddot{\mathbf{R}}_{i,A} = -\frac{1}{M_A} \nabla_A E_n(\mathbf{R}_i) \tag{7}$$

Here, n is the index of the occupied state, and M_A is the mass of nucleus A.

Nonadiabatic effects are accounted for by allowing each simulated trajectory to undergo a switch (or, "hop") from the current state into another state from among those included in the linear expansion 4, which then becomes the new current state for the given trajectory. The switches are imposed stochastically according to the criterion proposed by Tully. This algorithm is designed in such a way as to ensure that the number of trajectories occupying each state is proportional to its population as defined by $|a_j(t)|^2$, and to achieve this goal with the lowest possible number of switches.

In order to obtain a global picture of state populations in the ensemble of simulated trajectories, one also defines the so-called classical populations. Specifically, the classical population $P_j(t)$ of the j-th state is defined as the fraction of trajectories currently occupying that state:

$$P_j(t) = \frac{N_j(t)}{N_{\text{trajs}}} \tag{8}$$

Here, N_{trajs} is the number of simulated trajectories.

As an illustration of how the fewest switches surface hopping algorithm describes the feedback between the electronic and the nuclear dynamics, Figure S5 shows a schematic illustration of internal conversion in a two-state system. For the sake of simplicity, we are considering only a single simulated trajectory. The system starts out occupying the upper adiabatic state (state 2). The initial population of state 2 is unity, and that of state 1 is zero. Internal conversion takes place while the system is traversing an avoided crossing between states 1 and 2. In the vicinity of the avoided crossing, the two states exhibit strong nonadiabatic coupling, such that most of the population is transferred from state 2 to state 1. As a result, at one point, the system undergoes a switch from state 2 to state 1. Afterwards, it continues to evolve, but now the occupied state is state 1.

In the present study, the NAMD simulations were carried out with the use of an in-house "wrapper" program containing an interface to the electronic structure program Q-Chem.[61,62] At each time step of a simulated trajectory, the wrapper calls Q-Chem for the calculation of state energies, the gradient of the occupied state, and the NACVs between the states included in linear expansion 4. (The setup of the electronic structure calculations will be given in the following section.) Once these calculations are finished, the wrapper parses the output from

Figure S5: Schematic illustration of internal conversion within the framework of the fewest switches surface hopping algorithm. See text for details.

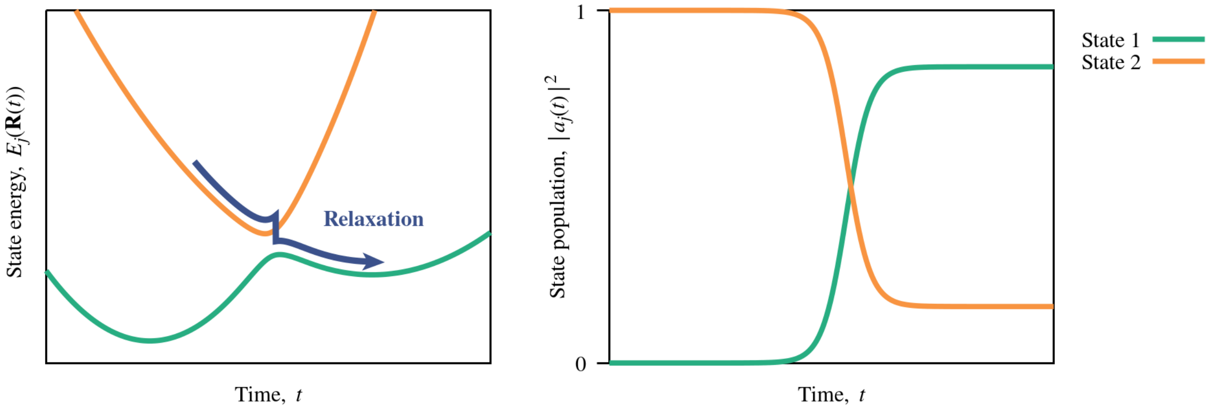

Q-Chem, and integrates the equations of motion (equations 5 and 7). For reference, the source code of the wrapper program is available for download from the repository Zenodo: https://doi.org/10.5281/zenodo.14910302

The dynamics of the nuclei (equation 7) was propagated with the use of the velocity Verlet integrator with a time step of 0.5 fs. The time-evolution of the wave function expansion coefficients (equation 5) was propagated with the fourth-order Runge-Kutta method with a time step of 0.001 fs. During the integration of equation 5, state energies and NACVs were interpolated linearly between successive classical steps. The simulated trajectories were propagated for a period of 600 fs.

The basic fewest switches surface hopping algorithm overestimates the coherence between the electronic states included in the linear expansion 4. For this reason, the time-evolution of the expansion coefficients $\{a_j(t)\}$ was corrected for decoherence via the scheme proposed by Granucci and Persico.[56] The correction constant was set to the value of $C = 0.1\ E_\mathrm{h}$ which was proposed by these authors.

Whenever the molecule hopped from one state to another, the nuclear velocities were rescaled in order to conserve the total energy. The rescaling was carried out according to the scheme proposed by Ferretti and co-workers:[63] if possible, the nuclear velocities were rescaled along the NACV between the initial and the final states. If rescaling the nuclear velocities along the NACV was not possible (because it could not satisfy energy conservation), the velocities were instead rescaled along the momentum vector. Lastly, in the event of a so-called "frustrated" hop – a situation where an upward hop cannot be imposed because the molecule has insufficient kinetic energy to reach the upper state – the nuclear velocities were left unchanged.

On another technical note, the phases of electronic wave functions calculated by a quantum chemistry program such as Q-Chem are set arbitrarily, and they can change from one time step to another. If the phase of one state k changes, but the phase of another state j does not, the NACV between these states will change direction (see equation 6). In order to correct for this effect, the wave function phases were tracked by calculating the normalized dot products

between NACVs calculated in successive time steps. When a phase change was detected, the NACV was multiplied by -1 from that point onward.

S3.2 Electronic Structure Calculations

As noted in the previous section, the NAMD method requires the "on-the-fly" calculation of state energies, gradients, and nonadiabatic couplings. Our initial intention was to calculate these quantities with MRSF-TDDFT, which we employed in the static calculations. However, we found that the ROKS reference calculation used in MRSF-TDDFT was prone to occasional convergence problems. While this was not a major issue in geometry optimizations, we expected that it might cause serious difficulties in the NAMD simulations, where the calculation must be done at each time step of every simulated trajectory. Even sporadic convergence failures would disrupt the simulations.

We reluctantly decided to cut the knot, and to use instead the conventional variant of SF-TDDFT with an unrestricted Kohn–Sham (UKS) reference, which we assumed would be easier to converge. In fact, with UKS we did not observe convergence problems per se – the reference UKS calculation always converged to tight tolerances. However, the analysis of the behavior of the UKS reference state revealed another issue: in the course of the NAMD simulations, its electronic structure occasionally underwent sudden changes. This effect and its consequences are discussed in more detail in Section S3.4 later on in this document. Fortunately, it mostly came into play *after* the molecule had undergone isomerization into the QC form. Thus, the early dynamics of the molecule towards the CI seam was not significantly affected. For this reason, the simulations still provide a realistic description of the photoinduced cycloaddition reaction; the underlying issue mainly affects the subsequent dynamics of the QC isomer.

The "on-the-fly" SF-TDDFT calculations were carried out with Q-Chem,[61,62] version 5.1.2. They were performed in the collinear approximation, and furthermore, the Tamm-Dancoff approximation[64] was imposed. Because the DTCAM functionals are not available in Q-Chem, we used instead the 50–50 functional.[7] This functional is based on the well-known B3LYP functional,[65,66] but it includes a larger fraction of HF exchange, which is required by the SF-TDDFT method. More specifically, its makeup is 50% HF + 8% Slater + 42% Becke for exchange, and 19% VWN + 81% LYP for correlation. The SCF convergence threshold was set to 10^{-9} E_h. The (99,590) grid was used.

As in the static MRSF-TDDFT calculations, we used the def2-SV(P) basis set. The NACVs between the states S_0, S_1, and S_2 were calculated analytically using the method of Zhang and Herbert.[67]

With regard to the accuracy of conventional SF-TDDFT, we will demonstrate below that the PESs predicted by that method are close to those obtained with MRSF-TDDFT. However, a separate issue is that, unlike MRSF-TDDFT, conventional SF-TDDFT gives rise to non-negligible spin contamination in the target states (as alluded to in Section S1 of this document). That is to say, the target states obtained with conventional SF-TDDFT are not eigenfunctions of the total spin-squared operator, $\hat{S}^2$. Furthermore, the expectation values of that operator deviate from the ideal values (0 for a singlet state, and 2 for a triplet state). Fortunately again, in the case of the compounds **I** and **II**, the problem is not too severe.

In order to demonstrate this point, we performed another test calculation, in which we used SF-TDDFT to re-calculate the energies of the low-lying electronic states of compound **I** along the reaction path for photoisomerization (see the main body of our paper, and also Section S1.2 in this document).

The results are shown in Figure S6. A point of note is that the SF-TDDFT calculation

Figure S6: Energies and $\langle S^2 \rangle$ values of the low-lying electronic states of compound **I** along the reaction path for photoinduced cycloaddition, as calculated at the SF-TDDFT level of theory.

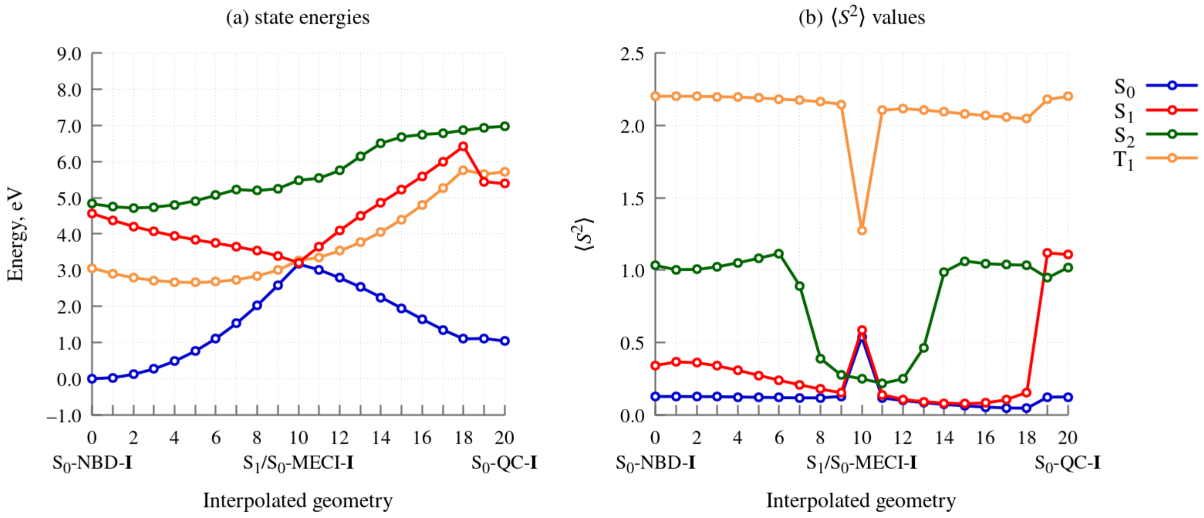

yields the low-spin component of state T_1 in addition to the singlet states. It can be seen that the potential energy curves predicted by conventional SF-TDDFT are very similar to those obtained with MRSF-TDDFT (see Figure S2 on page 7 in this document). As for spin contamination, its extent depends strongly on the electronic state in question, and on molecular geometry. State S_0 only shows appreciable spin contamination at the S_1/S_0-MECI structure. The occurrence of spin contamination near the S_1/S_0-MECI is apparently a result of artificial "mixing" between states S_0, S_1, and T_1, all of which are close in energy at that particular molecular geometry. State S_1 shows spin contamination near the S_1/S_0-MECI, but also at geometries close to the ground-state equilibrium geometry of the QC isomer (S_0-QC-**I**).

The behavior of the $\langle S^2 \rangle$ value of state S_2 is quite complex. Indeed, this state is significantly spin-contaminated near the ground-state equilibrium geometry of the NBD isomer (S_0-QC-**I**), with $\langle S^2 \rangle$ values close to unity. Although the spin contamination abates near the S_1/S_0-MECI structure, it later reappears near the ground-state equilibrium geometry of the QC isomer. The changes in the slope of the potential energy curve of state S_2, and the sudden changes in its $\langle S^2 \rangle$ value, indicate that the diabatic character of that state changes along the reaction coordinate.

The occurrence of spin contamination necessitates an automatic criterion to assign definite spin multiplicity (singlet or triplet) to the target states. We implemented the following state assignment scheme. At each classical time step of each trajectory, the wrapper had Q-Chem calculate the four target states with the lowest energies. The state with the highest $\langle S^2 \rangle$ value from among these four states was taken to be state T_1. The reason for that is that state T_1 is

expected to be always present among the four lowest target states. The other three target states were considered to be S_0, S_1, and S_2. A similar approach was used previously in Refs. 68–70.

S3.3 Initial Conditions

The initial conditions for the NAMD simulations were generated via the following procedure. Firstly, we optimized the ground-state equilibrium geometry of the NBD isomer of compound **II** at the DFT level. In this calculation, we used the restricted Kohn–Sham formalism. The remaining computational parameters were as specified in Section S3.2 of this document. We calculated the normal modes of the molecule numerically at the optimized geometry.

Afterwards, we sampled 2000 phase space points (which is to say, 2000 sets of nuclear positions and velocities) from the harmonic oscillator Wigner distribution. The sampling was performed with the use of the subprogram "initcond.pl" of Newton-X, version 1.4.0-2.[71–73] As discussed in Refs. 74–76, molecular dynamics simulations of molecules containing hydrogen atoms are prone to an artificial leakage of zero-point energy from high-frequency modes, and especially from the stretching modes of bonds to hydrogen atoms. In order to mitigate this problem, when generating the Wigner distribution we froze the highest 11 vibrational modes, which correspond to C–H stretching modes.

The 2000 geometries sampled from the Wigner distribution were used as the basis for the simulation of the absorption spectrum of compound **II**. This calculation was performed with the nuclear ensemble method.[77] A Gaussian line shape function was used with a variance of $\sigma^2 = 0.04\ \text{eV}^2$. The resulting spectrum is shown in Figure S7. Note that, due to the fact that the simulation only included states S_1 and S_2, the simulated spectrum only includes the first absorption band. At photon energies above around 5.5 eV, the calculated photoabsorption drops off to zero.

In order to represent photoexcitation with monochromatic light, we sampled a subset

Figure S7: Simulated absorption spectrum of compound **II**. $\sigma(E)$ is the photoabsorption cross section at photon energy E. The shaded area below the curve represents the energy interval from which initial conditions for the NAMD simulations were sampled.

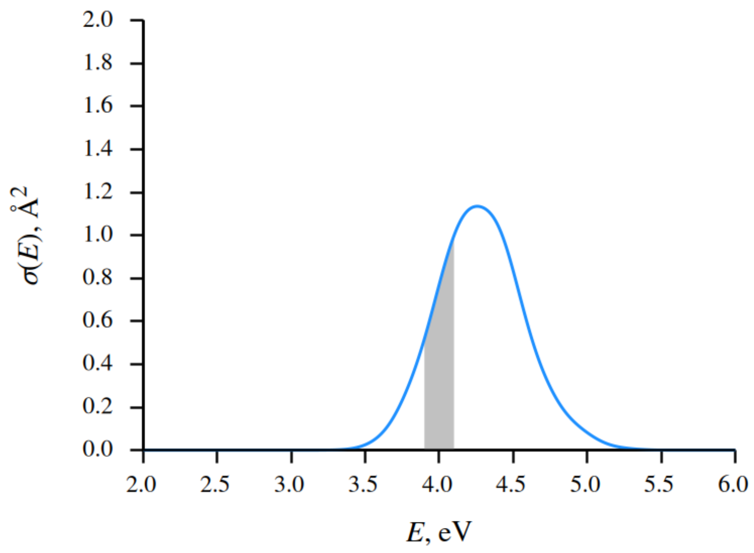

consisting of 100 phase space points for which the $S_0 \rightarrow S_1$ and/or the $S_0 \rightarrow S_2$ transition fell in the energy range of 3.9–4.1 eV. The phase space points to be included in this subset, and the initial electronic states (either S_1 or S_2), were sampled stochastically with probability proportional to the oscillator strength of the relevant transition. Each of the phase space points selected in this manner was used as the starting point for a single simulated trajectory.

S3.4 Analysis of UKS Reference State

One final issue that needs to be addressed is the behavior of the UKS reference state. Although the reference state is not directly used in the NAMD simulation, its electronic structure ultimately determines the electronic structures of the target states. Ideally, one would want the reference state to maintain the same diabatic character (for example, $^3\pi\pi^*$) across a wide range of molecular geometries. However, this is not the case for compound **II** studied by the NAMD simulations, and this has consequences for the corresponding simulation results.

We found that the easiest way to demonstrate this point is visually. Specifically, a convenient way to illustrate the electronic structure of the reference state is to plot its spin density, which is defined as the difference between the α and β electron densities. Accordingly, we generated animations of 10 representative NAMD trajectories in which the spin density of the UKS reference state at the given point in time is overlaid onto the molecular geometry. We have included these animations as part of the Electronic Supporting Information for this paper. The spin density of the UKS reference state is plotted in the form of isosurfaces with isovalues of $\pm 0.01\ e/{a_0}^3$. Red and blue isosurfaces, respectively, enclose regions with positive and negative values of the spin density,

At the early stage of each simulated trajectory, the reference state mostly maintains its diabatic character. Its spin density is delocalized over the π-bonding system, and its evolution in time is smooth and fairly slow. Informally speaking, the spin density is merely being dragged along with the nuclei.

In those trajectories where the molecule undergoes cycloaddition, this process is followed by intermittent sudden changes in the distribution of the spin density. The reference state switches back and forth between two types of solution, of which one corresponds to the spin density being localized on the norbornadiene moiety, and the other on the phenylethynyl ($C{\equiv}C{-}C_6H_5$) moiety.

As an alternative way of visualizing this effect, in Figure S8 on the following page we included a series of snapshots from a typical simulated trajectory which undergoes photoisomerization. In this case, ring closing took place at around $t = 200$ fs. After that, the character of the reference state changed repeatedly. One instance of this effect is seen at $t = 350$ fs, but there were also others at different times along this trajectory.

The sudden changes in the character of the reference state apparently introduce discontinuities in the PESs of the SF-TDDFT target states. As a result, the total energy in the NAMD simulation begins to drift upwards. This situation is only acceptable because the problem mainly manifests itself already *after* cycloaddition; energy conservation is generally good until after the molecule has undergone photoisomerization. For this reason, the key calculated quantities, such as the timescale and the quantum yield of photoisomerization, are not expected to be affected.

Figure S8: Series of snapshots from a typical simulated trajectory of compound **II** at intervals of 50 fs. The spin density of the UKS reference state is plotted in the form of isosurfaces with isovalues of $\pm 0.01\ e/{a_0}^3$.

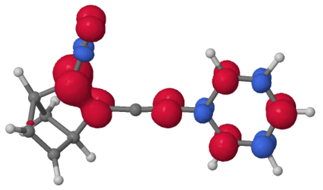
$t = 0$ fs

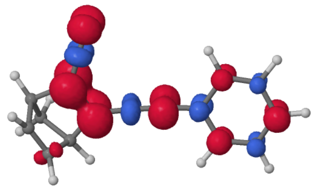
$t = 50$ fs

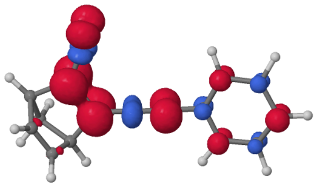
$t = 100$ fs

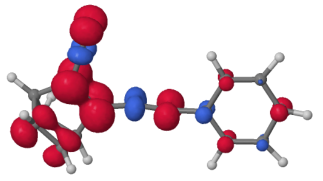
$t = 150$ fs

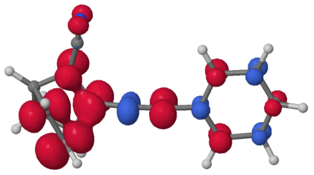
$t = 200$ fs

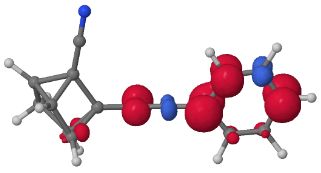
$t = 250$ fs

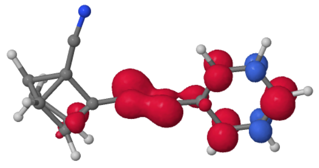
$t = 300$ fs

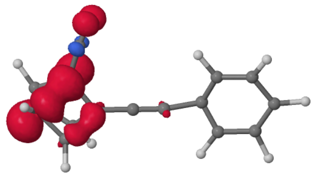
$t = 350$ fs

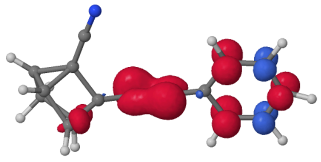
$t = 400$ fs

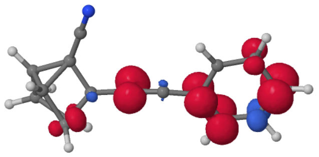
$t = 450$ fs

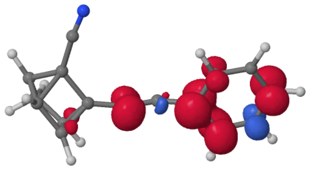
$t = 500$ fs

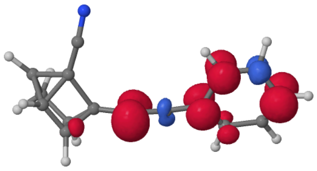
$t = 550$ fs

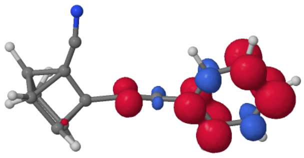
$t = 600$ fs

References

[1] Lee, S.; Filatov, M.; Lee, S.; Choi, C. H. Eliminating Spin-Contamination of Spin-Flip Time Dependent Density Functional Theory within Linear Response Formalism by the use of Zeroth-Order Mixed-Reference (MR) Reduced Density Matrix. *J. Chem. Phys.* **2018**, *149*, 104101. DOI: 10.1063/1.5044202

[2] Horbatenko, Y.; Lee, S.; Filatov, M.; Choi, C. H. Performance Analysis and Optimization of Mixed-Reference Spin-Flip Time-Dependent Density Functional Theory (MRSF-TDDFT) for Vertical Excitation Energies and Singlet–Triplet Energy Gaps. *J. Phys. Chem. A* **2019**, *123*, 7991–8000. DOI: 10.1021/acs.jpca.9b07556

[3] Horbatenko, Y.; Sadiq, S.; Lee, S.; Filatov, M.; Choi, C. H. Mixed-Reference Spin-Flip Time-Dependent Density Functional Theory (MRSF-TDDFT) as a Simple yet Accurate Method for Diradicals and Diradicaloids. *J. Chem. Theory Comput.* **2021**, *17*, 848–859. DOI: 10.1021/acs.jctc.0c01074

[4] Park, W.; Komarov, K.; Lee, S.; Choi, C. H. Mixed-Reference Spin-Flip Time-Dependent Density Functional Theory: Multireference Advantages with the Practicality of Linear Response Theory. *J. Phys. Chem. Lett.* **2023**, *14*, 8896–8908. DOI: 10.1021/acs.jpclett.3c02296

[5] Lee, S.; Park, W.; Choi, C. H. Expanding Horizons in Quantum Chemical Studies: The Versatile Power of MRSF-TDDFT. *Acc. Chem. Res.* **2025**, *58*, 208–217. DOI: 10.1021/acs.accounts.4c00640

[6] Krylov, A. I. Size-Consistent Wave Functions for Bond-Breaking: the Equation-of-Notion Spin-Flip Model. *Chem. Phys. Lett.* **2001**, *338*, 375–384. DOI: 10.1016/S0009-2614(01)00287-1

[7] Shao, Y.; Head-Gordon, M.; Krylov, A. I. The Spin-Flip Approach Within Time-Dependent Density Functional Theory: Theory and Applications to Diradicals. *J. Chem. Phys.* **2003**, *118*, 4807–4818. DOI: 10.1063/1.1545679

[8] Levchenko, S. V.; Krylov, A. I. Equation-of-Motion Spin-Flip Coupled-Cluster Model with Single and Double Substitutions: Theory and Application to Cyclobutadiene. *J. Chem. Phys.* **2004**, *120*, 175–185. DOI: 10.1063/1.1630018

[9] Krylov, A. I. Spin-Flip Equation-of-Motion Coupled-Cluster Electronic Structure Method for a Description of Excited States, Bond Breaking, Diradicals, and Triradicals. *Acc. Chem. Res.* **2006**, *39*, 83–91. DOI: 10.1021/ar0402006

[10] Casanova, D.; Krylov, A. I. Spin-Flip Methods in Quantum Chemistry. *Phys. Chem. Chem. Phys.* **2020**, *22*, 4326–4342. DOI: 10.1039/C9CP06507E

[11] Herbert, J.; Mandal, A. ChemRxiv. **2022**. Spin-Flip TDDFT for Photochemistry. DOI: 10.26434/chemrxiv-2022-gj75d

[12] Lee, S.; Shostak, S.; Filatov, M.; Choi, C. H. Conical Intersections in Organic Molecules: Benchmarking Mixed-Reference Spin–Flip Time-Dependent DFT (MRSF-TD-DFT) vs Spin–Flip TD-DFT. *J. Phys. Chem. A* **2019**, *123*, 6455–6462. DOI: 10.1021/acs.jpca.9b06142

[13] Baek, Y. S.; Lee, S.; Filatov, M.; Choi, C. H. Optimization of Three State Conical Intersections by Adaptive Penalty Function Algorithm in Connection

with the Mixed-Reference Spin-Flip Time-Dependent Density Functional Theory Method (MRSF-TDDFT). *J. Phys. Chem. A* **2021**, *125*, 1994–2006. DOI: 10.1021/acs.jpca.0c11294

[14] Shiozaki, T.; Győrffy, W.; Celani, P.; Werner, H.-J. Communication: Extended Multi-State Complete Active Space Second-Order Perturbation Theory: Energy and Nuclear Gradients. *J. Chem. Phys.* **2011**, *135*, 081106. DOI: 10.1063/1.3633329

[15] Vladimir Mironov, Konstantin Komarov, Jingbai Li, Igor Gerasimov, Hiroya Nakata, Mohsen Mazaherifar, Kazuya Ishimura, Woojin Park, Alireza Lashkaripour, Minseok Oh, Miquel Huix-Rotllant, Seunghoon Lee, and Cheol Ho Choi OpenQP: A Quantum Chemical Platform Featuring MRSF-TDDFT with an Emphasis on Open-Source Ecosystem. *J. Chem. Theory Comput.* **2024**, *20*, 9464–9477. DOI: 10.1021/acs.jctc.4c01117

[16] Komarov, K.; Oh, M.; Nakata, H.; Lee, S.; Choi, C. H. UMRSF-TDDFT: Unrestricted Mixed-Reference Spin-Flip-TDDFT. *J. Phys. Chem. A* **2024**, *128*, 9526–9537. DOI: 10.1021/acs.jpca.4c04521

[17] Park, W.; Lashkaripour, A.; Komarov, K.; Lee, S.; Huix-Rotllant, M.; Choi, C. H. Toward Consistent Predictions of Core/Valence Ionization Potentials and Valence Excitation Energies by MRSF-TDDFT. *J. Chem. Theory Comput.* **2024**, *20*, 5679–5694. DOI: 10.1021/acs.jctc.4c00640

[18] Komarov, K.; Park, W.; Lee, S.; Huix-Rotllant, M.; Choi, C. H. Doubly Tuned Exchange–Correlation Functionals for Mixed-Reference Spin-Flip Time-Dependent Density Functional Theory. J. Chem. Theory Comput. **2023**, *19*, 7671–7684. DOI: 10.1021/acs.jctc.3c00884

[19] Weigend, F.; Ahlrichs, R. Balanced Basis Sets of Split Valence, Triple Zeta valence and Quadruple Zeta Valence Quality for H to Rn: Design and Assessment of Accuracy. *Phys. Chem. Chem. Phys.* **2005**, *7*, 3297–3305. DOI: 10.1039/b508541a

[20] Murray, C. W.; Handy, N. C.; Laming, G. J. Quadrature Schemes for Integrals of Density Functional Theory. *Mol. Phys.* **1993**, *78*, 997–1014. DOI: 10.1080/00268979300100651.

[21] Ciminelli, C.; Granucci, G.; Persico, M. The Photoisomerization Mechanism of Azobenzene: A Semiclassical Simulation of Non-adiabatic Dynamics. *Chem. - Eur. J.* **2004**, *10*, 2327–2341. DOI: 10.1002/chem.200305415

[22] Keal, T. W.; Koslowski, A.; Thiel, W. Comparison of Algorithms for Conical Intersection Optimisation using Semiempirical Methods. *Theor. Chem. Acc.* **2007**, *118*, 837–844. DOI: 10.1007/s00214-007-0331-5

[23] Gaussian 09, Revision D.01. Frisch, M. J.; Trucks, G. W.; Schlegel, H. B.; Scuseria, G. E.; Robb, M. A.; Cheeseman, J. R.; Scalmani, G.; Barone, V.; Petersson, G. A.; Nakatsuji, H.; Li, X.; Caricato, M.; Marenich, A. V.; Bloino, J.; Janesko, B. G.; Gomperts, R.; Mennucci, B.; Hratchian, H. P.; Ortiz, J. V.; Izmaylov, A. F.; Sonnenber, J. L.; Williams-Young, D.; Ding, F.; Lipparini, F.; Egidi, F.; Goings, J.; Peng, B.; Petrone, A.; Henderson, T.; Ranasinghe, D.; Zakrzewsk, V. G.; Gao, J.; Rega, N.; Zheng, G.; Liang, W.; Hada, M.; Ehara, M.; Toyota, K.; Fukuda, R.; Hasegawa, J.; Ishida, M.; Nakajima, T.; Honda, Y.; Kitao, O.; Nakai, H; Vreven, T.; Throssell, K.; Montgomery, J. A., Jr.; Peralta, J. E.; Ogliaro, F.; Bearpark, M. J.; Heyd, J. J.; Brothers, E. N..; Kudin, K. N.;

Staroverov, V. N.; Keith, T. A.; Kobayashi, R.; Normand, J.; Raghavachari, K.; Rendell, A. P.; Burant, J. C.; Iyengar, S. S.; Tomasi, J.; Cossi, M.; Millam, J. M.; Klene, M; Adamo, C.; Cammi, R.; Ochterski, J. W.; Martin, R. L.; Morokuma, K.; Farkas, O.; Foresman, J. B.; Fox, D. J. Gaussian, Inc.: Wallingford, CT, 2016.

[24] Pulay, P.; Fogarasi, G.; Pang, F.; Boggs, J. E. Systematic ab Initio Gradient Calculation of Molecular Geometries, Force Constants, and Dipole-Moment Derivatives. *J. Am. Chem. Soc.* **1979**, *101*, 2550–2560. DOI: 10.1021/ja00504a009

[25] Schlegel, H. B. Optimization of Geometries and Transition Structures. *J. Comput. Chem.* **1982**, *3*, 214–218. DOI: 10.1002/jcc.540030212

[26] Fogarasi, G.; Zhou, X.; Taylor, P. W.; Pulay, P. The Calculation of ab Initio Molecular Geometries: Efficient Optimization by Natural Internal Coordinates and Empirical Correction by Offset Forces. *J. Am. Chem. Soc.* **1992**, *114*, 8191–8201. DOI: 10.1021/ja00047a032

[27] Pulay, P.; Fogarasi, G. Geometry Optimization in Redundant Internal Coordinates. *J. Chem. Phys.* **1992**, *96*, 2856–2860. DOI: 10.1063/1.462844

[28] Baker, J. Techniques for Geometry Optimization: A Comparison of Cartesian and Natural Internal Coordinates. *J. Comput. Chem.* **1993**, *14*, 1085–1100. DOI: 10.1002/jcc.540140910

[29] Peng, C.; Schlegel, H. B. Combining Synchronous Transit and Quasi-Newton Methods for Finding Transition States. *Isr. J. Chem.* **1993**, *33*, 449–454. DOI: 10.1002/ijch.199300051

[30] Peng, C.; Ayala, P. Y.; Schlegel, H. B.; Frisch, M. J. Using Redundant Internal Coordinates to Optimize Equilibrium Geometries and Transition States. *J. Comput. Chem.* **1996**, *17*, 49–56. DOI: 10.1002/(SICI)1096-987X(19960115)17:1<49::AID-JCC5>3.0.CO;2-0

[31] Li, X.; Frisch, M. J. Energy-Represented DIIS within a Hybrid Geometry Optimization Method. *J. Chem. Theory Comput.* **2006**, *2*, 835–839. DOI: 10.1021/ct050275a

[32] https://github.com/Open-Quantum-Platform/openqp/wiki/MRSF-TDDFT%20NACME, accessed on March 31, 2025.

[33] BAGEL, Brilliantly Advanced General Electronic-structure Library. http://www.nubakery.org under the GNU General Public License.

[34] Dunning, T. H. Gaussian Basis Sets for Use in Correlated Molecular Calculations. I. The Atoms Boron through Neon and Hydrogen. *J. Chem. Phys.* **1989**, *90*, 1007–1023. DOI: 10.1063/1.456153

[35] Weigend, F. A Fully Direct RI-HF Algorithm: Implementation, Optimised Auxiliary Basis Sets, Demonstration of Accuracy and Efficiency. *Phys. Chem. Chem. Phys.* **2002**, *4*, 4285–4291. DOI: 10.1039/B204199P

[36] Roos, B. O. The Complete Active Space Self-Consistent Field Method and its Applications in Electronic Structure Calculations. In *Advances in Chemical Physics: Ab Initio Methods in Quantum Chemistry Part 2.* Lawley, K. P., Ed.; John Wiley & Sons Ltd., 1987; pp 399–445.

[37] Finley, J.; Malmqvist, P.-A.; Roos, B. O.; Serrano-Andrés, L. The Multi-State CASPT2 Method. *Chem. Phys. Lett.* **1998**, *288*, 299–306. DOI: 10.1016/S0009-2614(98)00252-8

[38] Christiansen, O.; Koch, H.; Jørgensen, P. The Second-Order Approximate Coupled Cluster Singles and Doubles Model CC2. *Chem. Phys. Lett.* **1995**, *243*, 409–418. DOI: 10.1016/0009-2614(95)00841-Q

[39] Hellweg, A.; Grüna, S. A.; Hättig, C. Benchmarking the Performance of Spin-Component Scaled CC2 in Ground and Electronically Excited States. *Phys. Chem. Chem. Phys.* **2008**, *10*, 4119–4127. DOI: 10.1039/B803727B

[40] Tajti, A.; Szalay, P. G. Accuracy of Spin-Component-Scaled CC2 Excitation Energies and Potential Energy Surfaces. *J. Chem. Theory Comput.* **2019**, *15*, 5523–5531. DOI: 10.1021/acs.jctc.9b00676

[41] Grimme, S. Improved Second-Order Møller–Plesset Perturbation Theory by Separate Scaling of Parallel- and Antiparallel-Spin Pair Correlation Energies. *J. Chem. Phys.* **2003**, *118*, 9095–9102. DOI: 10.1063/1.1569242

[42] TURBOMOLE V7.4.0 2020, a development of University of Karlsruhe and Forschungszentrum Karlsruhe GmbH, 1989–2007, TURBOMOLE GmbH, since 2007; available from http://www.turbomole.com.

[43] Balasubramani, S. G.; Chen, G. P.; Coriani, S.; Diedenhofen, M.; Frank, M. S.; Franzke, Y. J.; Furche, F.; Grotjahn, R.; Harding, M. E.; Hättig, C. et al. TURBOMOLE: Modular Program Suite for *Ab Initio* Quantum-Chemical and Condensed-Matter Simulations. *J. Chem. Phys.* **2020**, *152*, 184107. DOI: 10.1063/5.0004635

[44] Haase, F.; Ahlrichs, R. Semi-direct MP2 Gradient Evaluation on Workstation Computers: The MPGRAD Program. *J. Comp. Chem.*, **1993**, *14*, 907–912. DOI: 10.1002/jcc.540140805

[45] Weigend, F.; Häser, M. RI-MP2: First Derivatives and Global Consistency. *Theor. Chem. Acc.*, **1997**, *97*, 331–340. DOI: 10.1007/s002140050269

[46] Hättig, C.; Weigend, F. CC2 Excitation Energy Calculations on Large Molecules Using the Resolution of the Identity Approximation. *J. Chem. Phys.*, **2000**, *113*, 5154–5161. DOI: 10.1063/1.1290013

[47] Köhn, A.; Hättig, C. Analytic Gradients for Excited States in the Coupled-Cluster Model CC2 Employing the Resolution-of-the-Identity Approximation. *J. Chem. Phys.*, **2003**, *119*, 5021–5036. DOI: 10.1063/1.1597635

[48] Hättig; Köhn, A. Transition Moments and Excited State First-Order Properties in the Second-Order Coupled Cluster Model CC2 Using the Resolution of the Identity Approximation. *J. Chem. Phys.* **2002**, *117*, 6939–6951. DOI: 10.1063/1.1506918

[49] Kendall, R. A.; Dunning, T. H.; Harrison, R. J. Electron Affinities of the First-Row Atoms Revisited. Systematic Basis Sets and Wave Functions. *J. Chem. Phys.* **1992**, *96*, 6796–6806. DOI: 10.1063/1.462569

[50] Weigend, F.; Köhn, A.; Hättig, C. Efficient Use of the Correlation Consistent Basis Sets in Resolution of the Identity MP2 Calculations. *J. Chem. Phys.* **2002**, *116*, 3175–3183. DOI: 10.1063/1.1445115

[51] Martin, R. L. Natural Transition Orbitals. *J. Chem. Phys.* **2003**, *118*, 4775–4777. DOI: 10.1063/1.1558471

[52] Plasser, F.; Wormit, M.; Dreuw, A. New Tools for the Systematic Analysis and Visualization of Electronic Excitations. I. Formalism. *J. Chem. Phys.* **2014**, *141*, 024106. DOI: 10.1063/1.4885819

[53] Tully, J. C.; Preston, R. K. Trajectory Surface Hopping Approach to Nonadiabatic Molecular Collisions: The Reaction of H^+ with D_2. *J. Chem. Phys.* **1971**, *55*, 562–572. DOI: 10.1063/1.1675788

[54] Tully, J. C. Molecular Dynamics with Electronic Transitions. *J. Chem. Phys.* **1990**, *93*, 1061–1071. DOI: 10.1063/1.459170

[55] Hammes-Schiffer, S.; Tully, J. C. Proton Transfer in Solution: Molecular Dynamics With Quantum Transitions. *J. Chem. Phys.* **1994**, *101*, 4657–4667. DOI: 10.1063/1.467455

[56] Granucci, G.; Persico, M. Critical Appraisal of the Fewest Switches Algorithm for Surface Hopping. *J. Chem. Phys.* **2007**, *126*, 134114. DOI: 10.1063/1.2715585

[57] Barbatti, M. Nonadiabatic Dynamics with Trajectory Surface Hopping Method. *WIREs Comput. Mol. Sci.* **2011**, *1*, 620–633. DOI: 10.1002/wcms.64

[58] Agostini, F.; Curchod, B. F. E. Different Flavors of Nonadiabatic Molecular Dynamics. *WIREs Comput. Mol. Sci.* **2019**, *9*, e1417. DOI: 10.1002/wcms.1417

[59] Quant, M.; Lennartson, A.; Dreos, A.; Kuisma, M.; Erhart, P.; Börjesson, K.; Moth-Poulsen, K. Low Molecular Weight Norbornadiene Derivatives for Molecular Solar-Thermal Energy Storage. *Chem. Eur. J.* **2016**, *22*, 13265–13274. DOI: 10.1002/chem.201602530

[60] Marx, D.; Hütter, J. Ab Initio Molecular Dynamics: Theory and Implementation. In *Modern methods and algorithms of quantum chemistry;* Grotendorst, J., Ed.; NIC Series, Vol. 1; John von Neumann Institute for Computing: Julich, Germany, 2000; pp 301–449.

[61] Krylov, A. I.; Gill, P. M. W.; Q-Chem: An Engine for Innovation. *WIREs Comput. Mol. Sci.* **2013**, *3*, 317–326. DOI: 10.1002/wcms.1122

[62] Shao, Y.; Gan, Z.; Epifanovsky, E.; Gilbert, A. T. B.; Wormit, M.; Kussmann, J.; Lange, A. W.; Behn, A.; Deng, J.; Feng, X.; Ghosh, D.; Goldey, M.; Horn, P. R.; Jacobson, L. D.; Kaliman, I.; Khaliullin, R. Z.; Kús, T.; Landau, A.; Liu, J.; Proynov, E. I.; Rhee, Y. M.; Richard, R.M.; Rohrdanz, M. A.; Steele, R. P.; Sundstrom, E. J.; Woodcock III, H. L.; Zimmerman, P. M.; Zuev, D.; Albrecht, B.; Alguire, E.; Austin, B.; Beran, G. J. O.; Bernard, Y.A.; Berquist, E.; Brandhorst, K.; Bravaya, K. B.; Brown, S. T.; Casanova, D.; Chang, C.-M.; Chen, Y.; Chien, S. H.; Closser, K. D.; Crittenden, D. L.; Diedenhofen, M.; DiStasio Jr., R. A.; Dop, H.; Dutoi, A. D.; Edgar, R. G.; Fatehi, S.; Fusti-Molnar, L.; Ghysels, A.; Golubeva-Zadorozhnaya, A.; Gomes, J.; Hanson-Heine, M. W. D.; Harbach, P. H. P.; Hauser, A. W.; Hohenstein, E. G.; Holden, Z. C.; Jagau, T.-C.; Ji, H.; Kaduk, B.; Khistyaev, K.; Kim, J.; Kim, J.; King, R. A.; Klunzinger, P.; Kosenkov, D.; Kowalczyk, T.; Krauter, C. M.; Lao, K. U.; Laurent, A.; Lawler, K. V.; Levchenko, S. V.; Lin, C. Y.; Liu, F.; Livshits, E.; Lochan, R. C.; Luenser, A.; P.Manohar, ; S.F.Manzer, ; S.-P.Mao, ; Mardirossian, N.; Marenich, A. V.; Maurer, S. A.; Mayhall, N. J.; Oana, C. M.; Olivares-Amaya, R.; O'Neill, D. P.; Parkhill, J. A.; Perrine, T. M.; Peverati, R.; Pieniazek, P. A.; Prociuk, A.; Rehn, D. R.; Rosta, E.; Russ, N. J.; Sergueev, N.; Sharada, S. M.; Sharmaa, S.;

Small, D.W.; Sodt, A.; Stein, T.; Stück, D.; Su, Y.-C.; Thom, A. J. W.; Tsuchimochi, T.; Vogt, L.; Vydrov, O.; Wang, T.; Watson, M. A.; Wenzel, J.; White, A.; Williams, C. F.; Vanovschi, V.; Yeganeh, S.; Yost, S. R.; You, Z.-Q.; Zhang, I.Y.; Zhang, X.; Zhou, Y.; Brooks, B. R.; Chan, G. K. L.; Chipman, D. M.; Cramer, C. J.; Goddard III, W.A.; Gordon, M.S.; Hehre, W.J.; Klamt, A.; Schaefer III, H. F.; Schmidt, M.W.; Sherrill, C. D.; Truhlar, D. G.; Warshel, A.; Xua, X.; Aspuru-Guzik, A.; Baer, R.; Bell, A. T.; Besley, N. A.; Chai, J.-D.; Dreuw, A.; Dunietz, B. D.; Furlani, T. R.; Gwaltney, S. R.; Hsu, C.-P.; Jung, Y.; Kong, J.; Lambrecht, D. S.; Liang, W.; Ochsenfeld, C.; Rassolov, V. A.; Slipchenko, L. V.; Subotnik, J. E.; VanVoorhis, T.; Herbert, J. M.; Krylov, A. I.; Gill, P. M. W.; Head-Gordon, M. Advances in Molecular Quantum Chemistry cContained in the Q-Chem 4 Program Package. *Mol. Phys.* **2015**, *113*, 184–215. DOI: 10.1080/00268976.2014.952696

[63] Ferretti, A.; Granucci, G.; Lami, A.; Persico, M.; Villani, G. Quantum Mechanical and Semiclassical Dynamics at a Conical Intersection. *J. Chem. Phys.* **1996**, *104*, 5517–5527. DOI: 10.1063/1.471791

[64] Hirata, S.; Head-Gordon, M. Time-Dependent Density Functional Theory within the Tamm–Dancoff Approximation. *Chem. Phys. Lett.* **1999**, *314*, 291–299. DOI: 10.1016/S0009-2614(99)01149-5

[65] Becke, A. D. Density-Functional Thermochemistry. III. The Role of Exact Exchange. *J. Chem. Phys.* **1993**, *98*, 5648–5652. DOI: 10.1063/1.464913

[66] Stephens, P. J.; Devlin, F. J.; Chabalowski, F.; Frisch, M. J. *Ab Initio* Calculation of Vibrational Absorption and Circular Dichroism Spectra Using Density Functional Force Fields. *J. Phys. Chem.* **1994**, *98*, 11623–11627. DOI: 10.1021/j100096a001

[67] Zhang, X.; Herbert, J. M. Analytic Derivative Couplings for Spin-Flip Configuration Interaction Singles and Spin-Flip Time-Dependent Density Functional Theory. *J. Chem. Phys.* **2014**, *141*, 064104. DOI: 10.1063/1.4891984

[68] Yue, L.; Liu, Y.; Zhu, C. Performance of TDDFT with and without Spin-Flip in Trajectory Surface Hopping Dynamics: *Cis–Trans* Azobenzene Photoisomerization. *Phys. Chem. Chem. Phys.* **2018**, *20*, 24123–24139. DOI: 10.1039/C8CP03851A

[69] Bil, A.; Kochman, M. A. Photoinduced Double Proton Transfer in the Glyoxal–Methanol Complex Revisited: The Role of the Excited States. *J. Chem. Theory Comput.* **2020**, *16*, 3273–3286. DOI: 10.1021/acs.jctc.0c00007

[70] Kochman, M A.; Gryber, T.; Durbeej, B.; Kubas, A. Simulation and Analysis of the Relaxation Dynamics of a Photochromic Furylfulgide. *Phys. Chem. Chem. Phys.* **2022**, *24*, 18103–18118. DOI: 10.1039/D2CP02143A

[71] Barbatti, M.; Granucci, G.; Persico, M.; Ruckenbauer, M.; Vazdar, M.; Eckert-Maksic, M.; Lischka, H. The On-the-Fly Surface-Hopping Program System Newton-X: Application to ab Initio Simulation of the Nonadiabatic Photodynamics of Benchmark Systems. *J. Photochem. Photobiol. A* **2007**, *190*, 228–240. DOI: 10.1016/j.jphotochem.2006.12.008

[72] Barbatti, M.; Ruckenbauer, M.; Plasser, F.; Pittner, J.; Granucci, G.; Persico, M.; Lischka, H. Newton-X: a Surface-Hopping Program for Nonadiabatic Molecular Dynamics. *WIREs Comput. Mol. Sci.* **2014**, *4*, 26–33. DOI: 10.1002/wcms.1158

[73] Barbatti, M.; Bondanza, M.; Crespo-Otero, R.; Demoulin, B.; Dral, P. O.; Granucci, G.; Kossoski, F.; Lischka, H.; Mennucci, B.; Mukherjee, S.; Pederzoli, M.; Persico, M.; Pinheiro Jr, M.; Pittner, J.; Plasser, F.; Gil, E. S.;Stojanovic, L. Newton-X Platform: New Software Developments for Surface Hopping and Nuclear Ensembles. *J. Chem. Theory Comput.* **2022**, *18*, 6851–6865. DOI: 10.1021/acs.jctc.2c00804

[74] Bowman, J. M.; Gazdy, B.; Sun, Q. A Method to Constrain Vibrational Energy in Quasiclassical Trajectory Calculations. *J. Chem. Phys.* **1989**, *91*, 2859–2862. DOI: 10.1063/1.456955

[75] Guo, Y.; Thompson, D. L.; Sewell, T. D. Analysis of the Zero-Point Energy Problem in Classical Trajectory Simulations. *J. Chem. Phys.* **1996**, *104*, 576–582. DOI: 10.1063/1.470853

[76] Mukherjee, S.; Barbatti, M. A Hessian-Free Method to Prevent Zero-Point Energy Leakage in Classical Trajectories. *J. Chem. Theory Comput.* **2022**, *18*, 4109–4116. DOI: 10.1021/acs.jctc.2c00216

[77] Crespo-Otero, R.; Barbatti, M. Spectrum Simulation and Decomposition with Nuclear Ensemble: Formal Derivation and Application to Benzene, Furan and 2-Phenylfuran. *Theor. Chem. Acc.* **2012**, *131*, 1237. DOI: 10.1007/s00214-012-1237-4
