# Supplementary material for: Simulating the Energy Capture Process in Push–Pull Norbornadiene-Quadricyclane Photoswitches
Source: J Phys Chem Lett. 2025 Apr 23;16(17):4315–25. doi: 10.1021/acs.jpclett.5c00634 (PMC12051204; doi:10.1021/acs.jpclett.5c00634)
Supplement: Supplementary file 5 — jz5c00634_si_005.pdf [file jz5c00634_si_005.pdf]

jz-2025-00634t.R1

Name: Peer Review Information for "Simulating the Energy Capture Process in Push-Pull Norbornadiene-Quadricyclane Photoswitches"

First Round of Reviewer Comments

Reviewer: 1

Comments to the Author

Michał and co-workers present a computational study on the photochemical ring-closing reactions of the push-pull norbornadiene systems. They performed state-of-the-art MRSF-TDDFT calculations to characterize the vertical excitation and carried out NAMD simulations using SF-TDDFT to study the excited-state dynamics. The manuscript is well-organized and written clearly. The conclusion on how push-pull substitution affects quantum yield is very interesting and will be helpful for the research community in developing MOST material. Thus, I recommend publishing this work after the authors address a few minor points.

1. The authors plot the NTO pairs for the S1 transition. I suggest plotting the density differences of the two NOTs to show the electron and hole, making it more straightforward to see the charge transfer characters.

2. The authors performed surface hopping dynamics. Were the non-adiabatic couplings explicitly computed or approximated with wavefunction overlap or surface curvature? How many trajectories were used in the discussion?

3. The author explained the overestimated quantum yield due to not accounting for the thermal cycloreversion. Why was this effect not included? Could this result from the PES computed at the SF-TDDFT level?

4. The author hypothesized that the large FC-MECI gap in the unsubstituted NBD could lead to low cycloaddition yield because the excessive energy could drive the reaction off the cycloaddition pathway. This is a very interesting point. Since the author had optimized

the MECIs, it will be interesting and useful to see the branching plane with the non-adiabatic coupling vectors. MRSF-TDDFT can compute the h-vector numerically.

5. The authors mentioned the ROHF convergence issue in MRSF-TDDFT, and they switched to SF-TDDFT for dynamics. Unfortunately, this is a fact, but it will improve in the future. How was the spin-contamination of SF-TDDFT calculated, and did the authors apply any state-tracking techniques to ensure the dynamics are running on the continues PES? I heard that Q-Chem provides spin-adapted SF-TDDFT. Why did they not use this approach?

Reviewer: 2

Comments to the Author

Dear Authors,

The work "Simulating the Energy Capture Process in Push-Pull

Norbornadiene-Quadricyclane Photoswitches" is sincerely welcome by this reviewer.

Main simulation outputs provide information on energies associated to intermediate states for the two molecules, which are used to define the reaction photo-switching path and the time evolution of the system. Meanwhile the former is compared with experimental data from absorption spectroscopy, the second one is not discussed in terms of comparison. Two minor comments emerge at this point:

a) Linear interpolation is used to define the reaction path. A short comment reflecting on how the results do not compromise the approach used would be welcome.

b) authors should comment on the availability of time dependent spectroscopic data related to the studied compounds, or promote explicitly the research on this subject.

Minor typo: double "the the" on page 5, line 12-13. As suggestion, several "Both these" could be shortened to "Both".

Yours,

Author's Response to Peer Review Comments:

Dr hab. Michał Andrzej
Kochman Institute of Physical
Chemistry of the Polish
Academy of Sciences Ruhr
University Bochum e-mail:
mkochman@ichf.edu.pl

Prof. Dr Bo Durbeej
Department of Physics, Chemistry and Biology (IFM),
Linköping University e-
mail: bodur@ifm.liu.se

Senior Editor
The Journal of Physical Chemistry Letters

Revision of Manuscript jz-2025-00634t:
Simulating the Energy Capture Process in Push-Pull
Norbornadiene-Quadricyclane Photoswitches

Dear Editor,

We are pleased to submit the revised version of our manuscript for consideration for publication in *The Journal of Physical Chemistry Letters.*

After taking into account the feedback from the Reviewers, we have addressed each of the Reviewers' points, and revised our manuscript as detailed in the attached file named Reply_to_Reviewers.pdf. For ease of reference, we are also attaching a marked manuscript file named Marked_Changes.pdf, in which the changes and corrections since the previously submitted version of the manuscript are highlighted in violet.

In closing, we would like to thank you for your time and consideration in handling the manuscript.

With best regards,

Michał Kochman

Michał Andrzej Kochman | Bo Durbeej

Michał Andrzej Kochman and Bo Durbeej

Response to Reviewers for Manuscript jz-2025-00634t: "Simulating the Energy Capture Process in Push-Pull Norbornadiene-Quadricyclane Photoswitches"

We would like to thank the Reviewers for their insightful and constructive feedback to our manuscript. Our responses to their questions and suggestions are listed below.

Section numbers and page numbers refer to the revised version of the manuscript.

The Reviewers' comments and questions are reproduced against a light grey background.

For ease of reference, some fragments of the revised manuscript are reproduced in boxes.

We are also attaching a marked manuscript file (Marked_Changes.pdf), in which the changes and corrections since the originally submitted version are highlighted in violet.

Response to Reviewer 1

Michał and co-workers present a computational study on the photochemical ring-closing reactions of the push-pull norbornadiene systems. They performed state-of-the-art MRSF-TDDFT calculations to characterize the vertical excitation and carried out NAMD simulations using SF-TDDFT to study the excited-state dynamics. The manuscript is well-organized and written clearly. The conclusion on how push-pull substitution affects quantum yield is very interesting and will be helpful for the research community in developing MOST material. Thus, I recommend publishing this work after the authors address a few minor points.

1. The authors plot the NTO pairs for the S1 transition. I suggest plotting the density differences of the two NOTs to show the electron and hole, making it more straightforward to see the charge transfer characters.

Author reply:

We agree it is a good idea to plot the hole and the particle densities. Accordingly, we have now performed these calculations, and discussed their results. For reasons of space, we have decided to place those plots and their discussion in Section S2 of the Supporting Information (SI), which also covers the setup of the second-order approximate coupled cluster singles and doubles (CC2) calculations. This addition to the SI is reproduced in the box below:

For either compound, the electronic structure of the lowest singlet excited state was visualized by plotting the most important natural transition orbital[1] (NTO) pair for the $S_0 \rightarrow S_1$ transition. These plots can be found in Figure 4 in the main body of our paper. As an alternative way of analyzing the electronic structures of states S_1 of the two compounds, we have also generated plots of the hole and the particle densities[2] for their respective $S_0 \rightarrow S_1$ transitions. The hole and particle densities and the NTOs are related through the following expressions:[2]

$$\text{hole density:} \quad \rho_{\mathrm{H}}(\mathbf{r}) = \sum_i \lambda_i \left(\psi_i^{\mathrm{H}}(\mathbf{r}) \right)^2 \tag{2}$$

$$\text{particle density:} \quad \rho_{\mathrm{P}}(\mathbf{r}) = \sum_i \lambda_i \left(\psi_i^{\mathrm{P}}(\mathbf{r}) \right)^2 \tag{3}$$

Here, $\psi_i^{\mathrm{H}}(\mathbf{r})$ and $\psi_i^{\mathrm{P}}(\mathbf{r})$ are, respectively, the i-th hole NTO and the i-th particle NTO associated with the $S_0 \rightarrow S_1$ transition, and λ_i is the eigenvalue corresponding to that NTO pair. For pragmatic reasons, when calculating the hole and the particle densities, we truncated

the sums in equations 2 and 3 after the last term with $\lambda_i > 0.005$.

The resulting hole and particle densities are plotted in Figure S4. It can be seen that, in compound **I**, the hole density is mainly localized in the π-bonding regions of the C2=C3 bond and, to a lesser extent, on the C5=C6 bond and on the phenyl and nitrile groups. Meanwhile, the particle density is predominantly localized in the π-bonding regions of the C2–Ph and the C3–CN bonds.

In compound **II**, in turn, the hole and the particle densities are partially delocalized onto the ethynyl (C≡C) bridge connecting atom C2 and the phenyl group.

Importantly, the topographies of the hole and the particle densities confirm that the lowest singlet states of compounds **I** and **II** do not show an appreciable intramolecular charge transfer character. Indeed, in both compounds, the particle density is largely localized in the same volume of space as the hole density. This is the hallmark of a locally excited state.

Figure S4: Hole and particle densities for the $S_0 \rightarrow S_1$ transitions of (a) compound **I** and (b) compound **II.** The densities are plotted in the form of isosurfaces with isovalues of $\pm 0.005\ {a_0}^{-3}$. The numbering of atoms in the norbornadiene moiety is marked in black.

(a) compound **I**

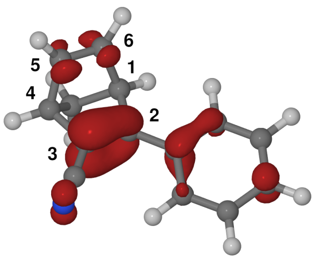

(i) hole density

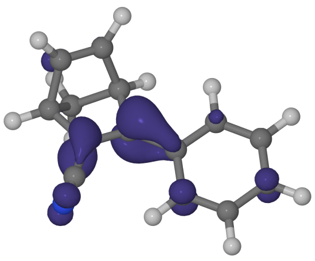

(ii) particle density

(b) compound **II**

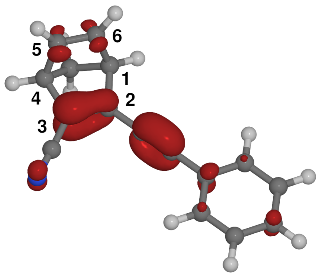

(i) hole density

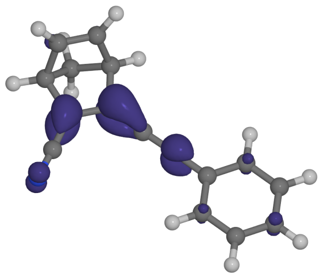

(ii) particle density

2. The authors performed surface hopping dynamics. Were the non-adiabatic couplings explicitly computed or approximated with wavefunction overlap or surface curvature? How many trajectories were used in the discussion?

Author Reply:

The calculation of the nonadiabatic coupling vectors is discussed in Section S3.2 of the SI, which covers the SF-TDDFT calculations that were integrated into the surface hopping simulations. Namely, the nonadiabatic coupling vectors were calculated analytically using the method of Zhang and Herbert,[3] which is implemented in Q-Chem.

We propagated a set of 100 trajectories. This information is provided on page 7 in the manuscript, and also in Section S3.3 of the SI, which describes the way that the initial conditions were set up.

We regret that some of the information asked about by the Reviewer had to be relegated to the SI. That was necessary in order to keep the main body of the manuscript within reasonable length. Because these simulation parameters are already provided either in the main body of the manuscript, or in the SI, we believe there is no need to make corrections to the manuscript in relation to this issue.

3. The author explained the overestimated quantum yield due to not accounting for the thermal cycloreversion. Why was this effect not included? Could this result from the PES computed at the SF-TDDFT level?

Author Reply:

Taking into account the thermal cycloreversion reaction of compound **II** would entail modeling the kinetic competition between ring opening and vibrational cooling – the dissipation of heat from the vibrationally excited QC isomer into the surrounding solvent. We feel that a simulation of this type would fall outside the scope of the present study, which is focused on the mechanism of the NBD$\rightarrow$QC photoisomerization itself. The subsequent dynamics of the photoproduct QC isomer is of less interest to us, even if it may potentially affect the photoisomerization quantum yield.

A separate issue is that the computational cost of running such a simulation seems prohibitive. The vibrational cooling of molecules in "hot" ground states resulting from non-radiative deactivation

takes place on timescales on the order of picoseconds.[4–6] (The exact timescale depends on the molecule in question, and on the surrounding medium.) In order to obtain meaningful data on the rate of vibrational cooling, we expect we would need to propagate the dynamics of the molecule for ca. 5 ps. Now, on the CPU type which we used which we used for most of our simulations (the Intel(R) Xeon(R) Platinum 8268 CPU running at 2.9 GHz), a single evaluation of the gradient of state S_0 of compound **II** with the SF-TDDFT method takes roughly 1 CPU hour. The cost of propagating a set of 100 trajectories for 5 ps with a time step of 0.5 fs would be on the order of 1,000,000 CPU hours. This is a very rough estimate, but the point still stands: this would be a very expensive simulation.

Of course, in such a simulation it would also be necessary to include the solvent, so as to account for heat dissipation. One way in which this could be achieved is by using the well-known hybrid quantum mechanics/molecular mechanics[7,8] (QM/MM) method. The inclusion of the solvent via the QM/MM approach is relatively inexpensive in terms of computing time; the main expense comes from the quantum-mechanical part of the overall calculation.

The Reviewer is correct to note that the overestimation of the quantum yield may also be due to other factors, such as the fact that the PESs were described using the SF-TDDFT method. We have now updated the relevant paragraph on page 19 in our manuscript to address the above-mentioned issues:

> The overestimation of the quantum yield may be partially due to the relatively short time frame of the simulations. The QC isomer is formed in the "hot" ground state. Some of the QC molecules may subsequently revert to the NBD isomer through a thermal cycloreversion reaction before they are able to dissipate heat into the surrounding solvent. This effect is not accounted for by the present simulations, firstly because the solvent is not included, and secondly because the simulation time is shorter than the timescale of vibrational cooling of molecules in hot ground states, which is on the order of picoseconds.[4–6] Other possible reasons for the overestimation of the quantum yield are the various approximations inherent in the simulations, such as the fact that the PESs are being calculated at the SF-TDDFT level of theory. Still, the discrepancy between simulation and experiment is, we believe, small enough that it does not cast doubt on the qualitative accuracy of the simulations.

In this paragraph, we have cited studies 4–6, which discuss the vibrational cooling processes of molecules in various environments.

4. The author hypothesized that the large FC-MECI gap in the unsubstituted NBD could lead to low cycloaddition yield because the excessive energy could drive the reaction off the cycloaddition pathway. This is a very interesting point. Since the author had optimized the MECIs, it will be interesting and useful to see the branching plane with the non-adiabatic coupling vectors. MRSF-TDDFT can compute the h-vector numerically.

Author Reply:

We agree that it will be informative to analyze the branching space vectors (the gradient difference vector – GDV – and the nonadiabatic coupling vector – NACV) at the S_1/S_0 minimum-energy conical intersection (MECI) geometries. The NACV in particular provides a useful connection between the static calculations (geometry optimizations and potential energy surface scans), and the nonadiabatic molecular dynamics (NAMD) simulations. Following the Reviewer's suggestion, we have now calculated and discussed the branching space vectors of compounds **I** and **II** at their respective S_1/S_0-MECI geometries.

On page 13 of the manuscript we have now introduced Figure 4, which shows the S_1-S_0 GDVs and NACVs as calculated at the MRSF-TDDFT level. For ease of reference, we are reproducing this Figure and the accompanying text below:

It is also of interest to inspect the branching space vectors (the gradient difference vector – GDV – and the nonadiabatic coupling vector – NACV) between the intersecting states at the S_1/S_0-MECI structures of compounds **I** and **II**. Accordingly, we calculated these vectors at the MRSF-TDDFT level of theory, and we show them in Figure 4. In both compounds, the branching space vectors show a similar structure, and they correspond to different deformations of the norbornadiene moiety: the GDV mainly involves to the motion of atom C3 away from atom C5, and the motion of atom C6 away from atom C2. The NACV, in turn, corresponds to the elongation of the C2=C3 and the C5=C6 bonds, accompanied by the contraction of the C2–C6 and the C3–C5 distances. In qualitative terms, the NACV is related to the change in the bonding pattern on going from the NBD isomer to the QC isomer.

In the picture afforded by the fewest switches surface hopping algorithm, internal conversion is driven by the movement of nuclei parallel, or antiparallel, to the NACV between the initial and the final state. (See Section S3.1 of the SI.) Thus, the structures the S_1-S_0 NACVs of compounds **I** and **II** indicate that, in both compounds, internal conversion from state S_1 to state S_0 is directly caused by ring closing.

Figure 4: Branching space vectors of (a) compound **I** and (b) compound **II** at their respective S_1/S_0-MECI structures.

(a) compound **I**

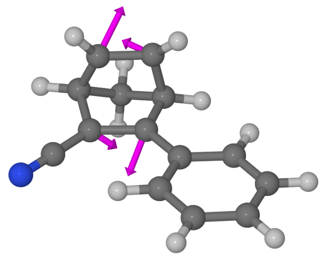

(i) GDV

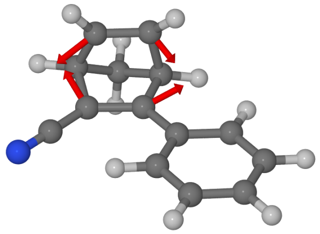

(ii) NACV

(b) compound **II**

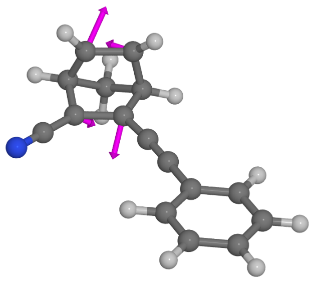

(i) GDV

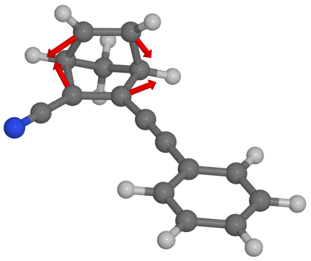

(ii) NACV

Moreover, technical details of the calculation of the NACVs have now been provided in Section S1.1 of the SI, which describes the setup of the MRSF-TDDFT calculations:

The S_1/S_0-MECI structures of compounds **I** and **II** were further characterized by plotting the branching space vectors – the gradient difference vector (GDV) and the nonadiabatic coupling vector (NACV) – between the intersecting states. As will be discussed in Section S3.1 in this document, internal conversion (population transfer between electronic states of the same multiplicity) is caused by the motion of nuclei parallel, or antiparallel, to the NACV between the initial and the final states. We took advantage of this property in order to identify the nuclear motions that bring about the $S_1 \rightarrow S_0$ internal conversion processes of compounds **I** and **II**.

The S_1-S_0 GDVs and NACVs of compounds **I** and **II** were calculated at their respective S_1/S_0 MECI geometries. For either compound, the GDV was calculated analytically as

$\nabla E(\mathrm{S}_1) - \nabla E(\mathrm{S}_0)$. Because the analytical calculation of the NACV is not available at the MRSF-TDDFT level, the calculations of the NACVs with that method were performed using the finite differences technique. Following the recommendation made in the OpenQP documentation,[9] the nuclear displacement for the purpose of calculating the NACV was set to 1×10^{-4} Å.

Note that in the NAMD simulations, we used the conventional variant of the SF-TDDFT method, and not the mixed-reference variant. In the course of the NAMD simulations, the NACVs were calculated analytically. See Section S3.2 in this document for more details.

Finally, the connection between the NACV and the nuclear motions responsible for internal conversion is now made clear in Section S3.1 of the SI, which provides an outline of the NAMD method:

The requirement that the electronic wave function $\Psi(\mathbf{r}, t; \mathbf{R}_i)$ is to satisfy the time-dependent electronic Schrödinger equation leads to the following system of coupled differential equations for the time-evolution of the expansion coefficients:

$$\mathrm{i}\,\hbar\,\dot{a}_k = \sum_j a_j(t)\left(\delta_{kj}\,E_k(\mathbf{R}_i) - \mathrm{i}\,\hbar\,\dot{\mathbf{R}}_i \cdot \mathbf{d}_{kj}(\mathbf{R}_i)\right) \tag{5}$$

where δ_{kj} denotes the Kronecker delta, $E_k(\mathbf{R})$ is the PES of the k-th adiabatic state, and $\mathbf{d}_{kj}(\mathbf{R})$ is the NACV between states k and j:

$$\mathbf{d}_{kj}(\mathbf{R}) = \langle\, \psi_k(\mathbf{r};\mathbf{R})\,|\,\nabla_{\mathbf{R}}\,|\,\psi_j(\mathbf{r};\mathbf{R})\,\rangle \tag{6}$$

A consequence of equation 5 is that internal conversion is driven by the motion of the nuclei parallel, or antiparallel, to the NACV between the initial and the final states. (For population transfer to take place between states k and j, the term $\dot{\mathbf{R}}_i \cdot \mathbf{d}_{kj}(\mathbf{R}_i)$ must be non-zero.) Thus, the calculation of the NACV provides a means of identifying the nuclear motions that are responsible for internal conversion.

5. The authors mentioned the ROHF convergence issue in MRSF-TDDFT, and they switched to SF-TDDFT for dynamics. Unfortunately, this is a fact, but it will improve in the future. How was the spin-contamination of SF-TDDFT calculated, and did the authors apply any state-tracking techniques to ensure the dynamics are running on the continues PES? I heard that Q-Chem provides spin-adapted SF-TDDFT. Why did they not use this approach?

Author Reply:

The way that we handled spin contamination in the SF-TDDFT calculations is described in Section S3.2 of the SI. For ease of reference, we are reproducing the relevant paragraph below:

> The occurrence of spin contamination necessitates an automatic criterion to assign definite spin multiplicity (singlet or triplet) to the target states. We implemented the following state assignment scheme. At each classical time step of each trajectory, the wrapper had Q-Chem calculate the four target states with the lowest energies. The state with the highest $\langle S^2 \rangle$ value from among these four states was taken to be state T_1. The reason for that is that state T_1 is expected to be always present among the four lowest target states. The other three target states were considered to be S_0, S_1, and S_2. A similar approach was used previously in Refs. 10–12.

This state assignment scheme is based on the observation that, even in the presence of spin contamination among the singlet states, state T_1 typically shows a much higher $\langle S^2 \rangle$ value than any of the singlet states. This was our experience in the present study, as well as in some previous studies of other systems.[11,12]. The advantages of this scheme are simplicity, and the fact that the state assignment is only a function of the $\langle S^2 \rangle$ values of the target states at the given molecular geometry. It does not depend on the prior history of the system. (As a side note, while researching the various state assignment schemes, we have now found that Yue and co-workers[10] have used essentially the same state assignment scheme in their study of the *cis*$\rightarrow$*trans* photoisomerization reaction of azobenzene. We have therefore added a citation of Ref. 10 in the relevant paragraph in the SI.)

It may be helpful to illustrate the functioning state assignment scheme on an example. On the following page, we are including the relevant output from an typical SF-TDDFT calculation for compound **II**. This particular calculation was performed at the ground-state equilibrium geometry as optimized at the 50-50/def2-SV(P) level of theory. Note that the "excitation energies" refer to spin-flipping excitations from the UKS reference state. In this case, "Excited state 2" shows the largest $\langle S^2 \rangle$ value from among the lowest four target states. It is therefore identified as state T_1. "Excited state 1" is state S_0 – the singlet ground state. "Excited state 3" and "Excited state 4" are considered to be states S_1 and S_2, respectively. Both these states show moderately severe spin contamination.

```
--------------------------------------------------
           SF-DFT Excitation Energies
(The first "excited" state might be the ground state)
--------------------------------------------------

Excited state   1: excitation energy (eV) =   -2.1575
Total energy for state  1:                 -669.93336883 au
   <S**2>     :  0.1273
   S(  2) -> S(  1) amplitude =  0.9732 alpha

Excited state   2: excitation energy (eV) =    0.6885
Total energy for state  2:                 -669.82877844 au
   <S**2>     :  2.1728
   D( 53) -> S(  1) amplitude = -0.3306
   D( 54) -> S(  1) amplitude =  0.1904
   S(  1) -> S(  1) amplitude =  0.5628 alpha
   S(  2) -> S(  2) amplitude =  0.6199 alpha
   S(  2) -> V(  2) amplitude =  0.1509 alpha
   S(  2) -> V(  3) amplitude =  0.2374 alpha

Excited state   3: excitation energy (eV) =    2.1606
Total energy for state  3:                 -669.77468315 au
   <S**2>     :  0.4769
   D( 56) -> S(  1) amplitude =  0.4412
   S(  1) -> S(  1) amplitude =  0.6207 alpha
   S(  2) -> S(  2) amplitude = -0.5514 alpha
   S(  2) -> V(  2) amplitude = -0.1701 alpha

Excited state   4: excitation energy (eV) =    2.3174
Total energy for state  4:                 -669.76891912 au
   <S**2>     :  0.9494
   D( 54) -> S(  1) amplitude = -0.3575
   D( 56) -> S(  1) amplitude =  0.7554
   S(  1) -> S(  1) amplitude = -0.3611 alpha
   S(  2) -> S(  2) amplitude =  0.2112 alpha
   S(  2) -> V(  2) amplitude =  0.1819 alpha
```

As pointed out by the Reviewer, Q-Chem does implement the spin-adapted variant of spin flip time-dependent density functional theory[13] (SA-SF-DFT). Unfortunately, however, analytic gradients are not available for this method, which makes it unsuited to molecular dynamics simulations. Moreover, the SA-SF-DFT method requires a restricted Kohn–Sham (ROKS) reference state, so it is likely to give rise to the same convergence difficulties as MRSF-TDDFT.

Another option is to use a state tracking algorithm, which aims to follow one or more target states over successive time steps. A number of algorithms of this type have been suggested in the literature; all are based on the idea that the electronic wave functions change slowly and continuously with the molecular geometry. Assuming one can reliably assign the spin multiplicities at the start of the simulation, it is then possible to follow the states during the subsequent dynamics of the system. (Also, state tracking can be used in geometry optimizations in order to follow the state of interest over successive optimization steps.)

Harabuchi and co-workers[14] used SF-TDDFT to simulate the excited-state relaxation dynamics of *cis*-stilbene. These authors implemented a state tracking scheme based on comparing the excitation vectors at different time steps.[14] However, they were careful to point out that, under certain circumstances, this algorithm performed poorly.[14] In particular, it became unable to follow the state of interest if that state interacted with another state for an extended period of time.[14]

Zhang and Herbert[13] proposed a state tracking scheme which relies on the comparison of transition densities at successive time steps. This algorithm was validated in the optimization of the S_1/S_0-MECI structure of ethylene.[13] Despite this success, Zhang and Herbert predicted that their algorithm may potentially fail in some situations, including in NAMD simulations.[13] In a later study of the excited-state dynamics of the protonated Schiff base model PSB3,[15] these authors employed a different approach. Namely, when solving the SF-TDDFT eigenvalue equations, they imposed a constraint on the excitation vectors which was intended to reduce spin contamination. Afterwards, they performed state assignment on the basis of the $\langle S^2 \rangle$ values.

Another state tracking algorithm was formulated by Closser and co-workers.[16] It is based on the comparison of attachment and the detachment densities[17] over successive time steps.[16] The Reviewer is correct to point out that this algorithm is implemented in the program Q-Chem. However, to the best of our knowledge, no data is available on the performance of this algorithm in the context of SF-TDDFT. It was originally developed for use in molecular dynamics simulations of electronically excited helium clusters[16] with the (spin-conserving) configuration interaction singles[18] (CIS) method, which does not suffer from spin contamination.

Yet another algorithm of this type was devised by García and co-workers.[19] In this case, the similarity between excited-state wave functions at successive molecular geometries is quantified by calculating overlaps between the corresponding NTOs.[19] We are not aware of this scheme having been applied in SF-TDDFT calculations.

There seems to be no definitive evidence to indicate that any of the state tracking algorithms proposed in the literature is superior to the simpler approach, in which the state assignment is based on the $\langle S^2 \rangle$ values. On the other hand, in the specific case of compound **II**, there is a strong argument against using a state tracking scheme: once the molecule is in a QC-like geometry, the UKS reference state intermittently flips between two types of solution. (See Section S3.4 of the SI.) Whenever that happens, the electronic structures of the SF-TDDFT target states will also change sharply from one time step to the next. This is likely to complicate efforts to follow the target states.

In summary, we believe that our approach is justified, and the alternative of using a state tracking algorithm is not necessarily better. Accordingly, we have not made changes to our simulations, or to the manuscript, in relation to this issue.

Response to Reviewer 2

> The work "Simulating the Energy Capture Process in Push-Pull Norbornadiene-Quadricyclane Photoswitches" is sincerely welcome by this reviewer.
>
> Main simulation outputs provide information on energies associated to intermediate states for the two molecules, which are used to define the reaction photo-switching path and the time evolution of the system. Meanwhile the former is compared with experimental data from absorption spectroscopy, the second one is not discussed in terms of comparison. Two minor comments emerge at this point:
>
> a) Linear interpolation is used to define the reaction path. A short comment reflecting on how the results do not compromise the approach used would be welcome.

Author Reply:

Regarding the way that the reaction paths were set up, we concede that generating them through linear interpolation in internal coordinates (LIIC) introduces a certain element of arbitrariness. This is because the resulting reaction path depends on the choice of internal coordinate system. However, in practice LIIC tends to provide physically reasonable reaction paths. By construction, the resulting reaction paths are smooth, in the sense that the molecular geometry varies continuously (linearly) along the reaction path. The main advantage is, of course, that one avoids having to optimize the reaction path, which can be a difficult proposition. Indeed, this approach is quite popular in the computational chemistry community – for example, it has been used in such studies as Refs. 20–26. (This list is not exhaustive, it is merely meant as an illustration of the widespread use of this technique.)

The interpolated reaction paths for compounds **I** and **II** are included in the SI for this manuscript. (They can be found in the ZIP file which also contains the input and output files from geometry optimizations and potential energy surface scans.) This will enable other researchers to view the reaction paths, and to reproduce the potential energy surface scans.

<u>In order to clarify the situation, on page 9 of the manuscript, we have now included a note which explains the fact that the interpolated reaction paths depend on the choice of the coordinate system:</u>

> We note here that using the LIIC procedure introduces a certain element of arbitrariness. This is because an interpolated reaction path depends on the choice of the internal coordinate system. Still, in practice LIIC tends to provide physically reasonable reaction paths. By

definition, the interpolated reaction paths are smooth, in the sense that the molecular geometry varies continuously (linearly) along the reaction path. The main advantage is, of course, that one avoids having to optimize the reaction path, which can be a difficult proposition. Indeed, this approach is quite popular in the computational chemistry community – for example, it has been used in such studies as Refs. 20–26. (This list is not exhaustive, it is merely meant as an illustration of the widespread use of this technique.) In order to demonstrate that the reaction paths that we have generated are physically reasonable, and also to enable other researchers to reproduce our results, we have included them as part of the SI.

b) authors should comment on the availability of time dependent spectroscopic data related to the studied compounds, or promote explicitly the research on this subject.

Author Reply:

We agree that the simulation results presented in the manuscript should be discussed in light of the available time-resolved spectroscopic data. To this end, we have added the following paragraphs on pages 19–20 of the manuscript:

A more direct comparison of the simulation results to experimental data is complicated by the fact that the NBD→QC photoisomerization reactions of compounds **I** and **II** have not been characterized with the use of time-resolved spectroscopic methods, such as transient absorption (TA) spectroscopy. To the best of our knowledge, the only study to date to have investigated the photochemistry of a push-pull NBD derivative with time-resolved methods was the work by Alex and co-workers.[27] These authors used TA spectroscopy to follow the photorelaxation processes of the NBD and the QC isomers of the photoswitch in which **R2** is a 4-(*N*,*N*-dimethylamino)phenyl group (p-C_6H_4–$N(CH_3)_2$), **R3** is a methyl carboxylate group (CO_2–CH_3), and **R5** and **R6** are hydrogen atoms.

However, the relaxation mechanism of the NBD isomer of that compound is substantially different from what our present simulations predict for compounds **I** and **II**. More specifically, in NBD isomer of the compound studied by Alex et al., the lowest singlet excited state has substantial ICT character.[27] This is attributable to strong electron-donating ability of the 4-(*N*,*N*-dimethylamino)phenyl group. Following the irradiation of the lowest photoabsorption band of the compound studied by Alex et al. in acetonitrile solution, the ICT state shows a relatively long lifetime of ca. 350 ps.[27]

Minor typo: double "the the" on page 5, line 12-13. As suggestion, several "Both these" could be shortened to "Both".

Author Reply:

We would like to thank the Reviewer for pointing out these grammar and stylistic issues. We have now corrected them.

Other Corrections

The various corrections and additions to the manuscript have increased its length. In order to keep the manuscript within reasonable length, we have decided to remove Figure 2 from the introductory section. This Figure served only to illustrate previous work by other authors and, as such, it was not absolutely essential to the manuscript. Its removal does not affect the findings of our manuscript in any way.

Very recently, Cooper and-coworkers have published a study[28] on the photochemistry of unsubstituted norbornadiene. This paper is relevant to the introductory section of our manuscript. Accordingly, we cited that paper in the introductory section. The conclusions and findings of our manuscript are not affected.

Following a request from the Editorial Office, we have amended the caption of Figure 5 in such a way that it explicitly refers to panels (a) and (b). This is a trivial formatting issue, and the findings and conclusions of the manuscript are not affected in any way. The corrected Figure appears as follows:

Figure 5: Dominant NTO pairs for the $S_0 \rightarrow S_1$ transitions of (a) compound **I** and (b) compound **II**, plotted in the form of isosurfaces with isovalues of $\pm 0.05\ {a_0}^{-3/2}$. The eigenvalue (λ_i) for each occupied-virtual NTO pair is given in terms of a percentage contribution.

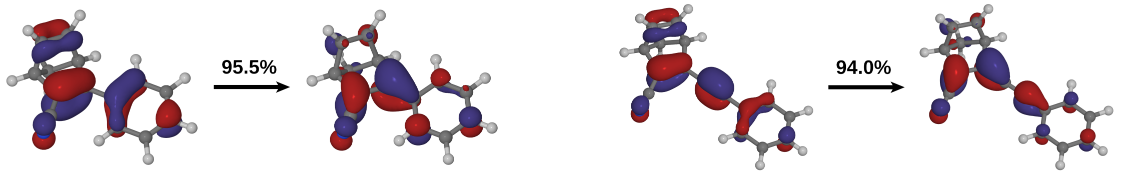

(a) compound **I**

(b) compound **II**

References

[1] Martin, R. L. Natural Transition Orbitals. *J. Chem. Phys.* **2003**, *118*, 4775–4777. DOI: 10.1063/1.1558471

[2] Plasser, F.; Wormit, M.; Dreuw, A. New Tools for the Systematic Analysis and Visualization of Electronic Excitations. I. Formalism. *J. Chem. Phys.* **2014**, *141*, 024106. DOI: 10.1063/1.4885819

[3] Zhang, X.; Herbert, J. M. Analytic Derivative Couplings for Spin-Flip Configuration Interaction Singles and Spin-Flip Time-Dependent Density Functional Theory. *J. Chem. Phys.* **2014**, *141*, 064104. DOI: 10.1063/1.4891984

[4] Pecourt, J.-M. L.; Peon, J.; Kohler, B. DNA Excited-State Dynamics: Ultrafast Internal Conversion and Vibrational Cooling in a Series of Nucleosides. *J. Am. Chem. Soc.* **2001**, *123*, 10370–10378. DOI: 10.1021/ja0161453

[5] Siewertsen, R.; Renth, F.; Temps, F.; Sönnichsen, F. Parallel Ultrafast *E–C* Ring Closure and *E–Z* Isomerisation in a Photochromic Furylfulgide Studied by Femtosecond Time-Resolved Spectroscopy. *Phys. Chem. Chem. Phys.* **2009**, *11*, 5952–5961. DOI: 10.1039/B821344E

[6] Ventura, E.; do Monte, S. A.; do Casal, M. T.; Pinheiro, M.; Toldo, J. M.; Barbatti, M. Modeling the Heating and Cooling of a Chromophore after Photoexcitation. *Phys. Chem. Chem. Phys.* **2022**, *24*, 9403–9410. DOI: 10.1039/D2CP00686C

[7] Lin, H.; Truhlar, D. G. QM/MM: What Have We Learned, Where Are We, and Where Do We Go From Here? *Theor. Chem. Acc.* **2007**, *117*, 185–199. DOI: 10.1007/s00214-006-0143-z

[8] Senn, H. M.; Thiel, W. QM/MM Methods for Biomolecular Systems. *Angew. Chem. Int. Ed.* **2009**, *48*, 1198–1229. DOI: 10.1002/anie.200802019

[9] https://github.com/Open-Quantum-Platform/openqp/wiki/MRSF-TDDFT%20NACME, accessed on March 31, 2025.

[10] Yue, L.; Liu, Y.; Zhu, C. Performance of TDDFT with and without Spin-Flip in Trajectory Surface Hopping Dynamics: *Cis–Trans* Azobenzene Photoisomerization. *Phys. Chem. Chem. Phys.* **2018**, *20*, 24123–24139. DOI: 10.1039/C8CP03851A

[11] Bil, A.; Kochman, M. A. Photoinduced Double Proton Transfer in the Glyoxal–Methanol Complex Revisited: The Role of the Excited States. *J. Chem. Theory Comput.* **2020**, *16*, 3273–3286. DOI: 10.1021/acs.jctc.0c00007

[12] Kochman, M A.; Gryber, T.; Durbeej, B.; Kubas, A. Simulation and Analysis of the Relaxation Dynamics of a Photochromic Furylfulgide. *Phys. Chem. Chem. Phys.* **2022**, *24*, 18103–18118. DOI: 10.1039/D2CP02143A

[13] Zhang, X.; Herbert, J. M. Spin-Flip, Tensor Equation-of-Motion Configuration Interaction with a Density-Functional Correction: A Spin-Complete Method for Exploring Excited-State Potential Energy Surfaces. *J. Chem. Phys.* **2015**, *143*, 234107. DOI: 10.1063/1.4937571

[14] Harabuchi, Y.; Keipert, K.; Zahariev, F.; Taketsugu, T.; Gordon, M. S. Dynamics Simulations with Spin-Flip Time-Dependent Density Functional Theory: Photoisomerization and Photocyclization Mechanisms of cis-Stilbene in $\pi\pi^*$ States. *J. Phys. Chem. A* **2014**, 118, 11987–11998. DOI: 10.1021/jp5072428

[15] Zhang, X.; Herbert, J. M. Nonadiabatic Dynamics with Spin-Flip vs Linear-Response Time-Dependent Density Functional Theory: A Case Study for the Protonated Schiff Base $C_5H_6NH_2^+$. *J. Chem. Phys.* **2021**, *155*, 124111. DOI: 10.1063/5.0062757

[16] Closser, K. D.; Gessner, O.; Head-Gordon, M. Simulations of the Dissociation of Small Helium Clusters with *ab Initio* Molecular Dynamics in Electronically Excited States. *J. Chem. Phys.* **2014**, *140*, 134306. DOI: 10.1063/1.4869193

[17] Head-Gordon, M.; Grana, A. M.; Maurice, D.; White, C. A. Analysis of Electronic Transitions as the Difference of Electron Attachment and Detachment Densities. *J. Phys. Chem.* **1995**, *99*, 14261–14270. DOI: 10.1021/j100039a012

[18] Foresman, J. B.; Head-Gordon, M.; Pople, J. A.; Frisch, M. J. Toward a Systematic Molecular Orbital Theory for Excited States. *J. Phys. Chem.* **1992**, *96*, 135–149. DOI: 10.1021/j100180a030

[19] García, J. S.; Boggio-Pasqua, M.; Ciofini, I.; Campetella, M. Excited State Tracking During the Relaxation of Coordination Compounds. *J. Comp. Chem.* **2019**, *40*, 1420–1428. DOI: 10.1002/jcc.25800

[20] Barbatti, M.; Vazdar, M.; Aquino, A. J. A.; Eckert-Maksić, M.; Lischka, H. The Nonadiabatic Deactivation Paths of Pyrrole. *J. Chem. Phys.* **2006**, *125*, 164323. DOI: 10.1063/1.2363376

[21] Nakayama, A.; Harabuchi, Y.; Yamazaki, S.; Taketsugua, T. Photophysics of Cytosine Tautomers: New Insights Into the Nonradiative Decay Mechanisms from MS-CASPT2 Potential Energy Calculations and Excited-State Molecular Dynamics Simulations. *Phys. Chem. Chem. Phys.* **2013**, *15*, 12322–12339. DOI: 10.1039/C3CP51617B

[22] Plasser, F.; Crespo-Otero, R.; Pederzoli, M.; Pittner, J.; Lischka, H.; Barbatti, M. Surface Hopping Dynamics with Correlated Single-Reference Methods: 9H-Adenine as a Case Study. *J. Chem. Theory Comput.* **2014**, *10*, 1395–1405. DOI: 10.1021/ct4011079

[23] Stojanović, L.; Bai, S.; Nagesh, J.; Izmaylov, A. F.; Crespo-Otero, R.; Lischka, H.; Barbatti, M. *Molecules* **2016**, *21*, 1603. DOI: 10.3390/molecules21111603

[24] Gao, Y.-H.; Chang, X.-P.; Liu, X.-Y.; Li, Q.-S.; Cui, G.; Thiel, W. Excited-State Decay Paths in Tetraphenylethene Derivatives. *J. Phys. Chem. A* **2017**, *121*, 2572–2579. DOI: 10.1021/acs.jpca.7b00197

[25] Marsili, E.; Prlj, A.; Curchod, B. F. E. Caveat When Using ADC(2) for Studying the Photochemistry of Carbonyl-Containing Molecules. *Phys. Chem. Chem. Phys.* **2021**, *23*, 12945–12949. DOI: 10.1039/D1CP02185K

[26] Valverde, D.; Mai, S.; Canuto, S.; Borin, A. C.; González, L. Ultrafast Intersystem Crossing Dynamics of 6-Selenoguanine in Water. *JACS Au* **2022**, *2*, 1699–1711. DOI: 10.1021/jacsau.2c00250

[27] Alex, W.; Lorenz, P.; Henkel, C.; Clark, T.; Hirsch, A.; Guldi, D. M. Solar Energy Storage: Competition between Delocalized Charge Transfer and Localized Excited States in the Norbornadiene to Quadricyclane Photoisomerization. *J. Am. Chem. Soc.* **2022**, *144*, 153–162. DOI: 10.1021/jacs.1c04322

[28] Cooper, J. C.; Brown, C. Y. Z.; Kára, J.; Kirrander, A. Photoexcited Dynamics of the Valence States of Norbornadiene. *J. Chem. Phys.* **2025**, *162*, 094102. DOI: 10.1063/5.0246270
